# Effectiveness of a Community-Based Structured Physical Activity Program for Adults With Type 2 Diabetes: A Randomized Clinical Trial

**DOI:** 10.1001/jamanetworkopen.2022.47858

**Published:** 2022-12-21

**Authors:** Aishee B. Mukherji, Di Lu, FeiFei Qin, Haley Hedlin, Neil M. Johannsen, Sukyung Chung, Yukari Kobayashi, Francois Haddad, Cynthia Lamendola, Marina Basina, Ruth Talamoa, Jonathan Myers, Latha Palaniappan

**Affiliations:** 1 Division of Primary Care and Population Health, Stanford University School of Medicine, Stanford, California; 2 Quantitative Sciences Unit, Stanford University School of Medicine, Stanford, California; 3 Pennington Biomedical Research Center, Louisiana State University, Baton Rouge; 4 Division of Cardiovascular Medicine, Stanford University School of Medicine, Stanford, California; 5 Stanford Cardiovascular Institute, Stanford, California; 6 Division of Endocrinology, Stanford University School of Medicine, Stanford, California; 7 Cardiology Division, VA Palo Alto Health Care System, Stanford University, Palo Alto, California; 8 Division of General Medical Disciplines, Department of Medicine, Stanford University School of Medicine, Stanford, California

## Abstract

**Importance:**

The efficacy of physical activity interventions among individuals with type 2 diabetes has been established; however, practical approaches to translate and extend these findings into community settings have not been well explored.

**Objective:**

To test the effectiveness of providing varying frequencies of weekly structured exercise sessions to improve diabetes control.

**Design, Setting, and Participants:**

The IMPACT (Initiate and Maintain Physical Activity in Communities Trial) study was a controlled randomized clinical trial (randomization occurred from October 2016 to April 2019) that included a 6-month, structured exercise intervention either once or thrice weekly vs usual care (UC; advice only). The exercise intervention was conducted at community-based fitness centers. Follow-up visits were conducted in a university research clinic. Participants included adults with type 2 diabetes (hemoglobin A_1c _[HbA_1c_] 6.5%-13.0%, not taking insulin, and no precluding health issues). Data analysis was performed from January to April 2022.

**Interventions:**

A once-weekly structured exercise group, a thrice-weekly structured exercise group, or UC.

**Main Outcomes and Measures:**

The primary outcome was HbA_1c_ at 6 months.

**Results:**

A total of 357 participants (143 women [40.1%]) with a mean (SD) age of 57.4 (11.1) years were randomized (119 each to the UC, once-weekly exercise, and thrice-weekly exercise groups). There was no significant difference in HbA_1c_ change by study group in the intention-to-treat analysis at 6 months. Specifically, HbA_1c_ changed by −0.23% (95% CI, −0.48% to 0.01%) in the thrice-weekly exercise group and by −0.16% (95% CI, −0.41% to 0.09%) in the once-weekly exercise group. A total of 62 participants (52.1%) in the once-weekly exercise group and 56 participants (47.1%) in the thrice-weekly exercise group were at least 50% adherent to the assigned structured exercise regimen and were included in the per-protocol analysis. Per-protocol analysis showed that HbA_1c_ changed by −0.35% (95% CI, −0.60% to −0.10%; *P* = .005) at 3 months and by −0.38% (95% CI, −0.65% to −0.12%; *P* = .005) at 6 months in the thrice-weekly exercise group compared with UC. There was no significant decrease in HbA_1c_ in the once-weekly exercise group. The exercise intervention was effective in improving self-reported minutes of metabolic equivalent tasks per week for participants in the thrice-weekly exercise group (both overall and per protocol).

**Conclusions and Relevance:**

Although the intervention was not effective in the intention-to-treat analysis, participants in the thrice-weekly exercise group who attended at least 50% of the sessions during the 6-month exercise intervention program improved HbA_1c_ levels at 6 months. Future efforts should focus on improving adherence to thrice-weekly structured exercise programs to meet exercise guidelines.

**Trial Registration:**

ClinicalTrials.gov Identifier: NCT02061579

## Key Points

**Question:**

What is the relative effectiveness of offering structured exercise sessions in improving diabetes control?

**Findings:**

In this 3-group randomized clinical trial, 357 adults with type 2 diabetes were advised to follow American Diabetes Association physical activity guidelines and randomized to usual care or once-weekly vs thrice-weekly structured exercise. There was no significant difference in hemoglobin A_1c_ in the intention-to-treat analysis, and hemoglobin A_1c_ was lowered only for participants in the thrice-weekly structured exercise program who attended at least 50% of the recommended exercise sessions.

**Meaning:**

These findings suggest that future efforts should focus on improving adherence to thrice-weekly structured exercise programs to meet exercise guidelines.

## Teaser

This randomized clinical trial examines the effectiveness of providing varying frequencies of weekly structured exercise sessions to improve diabetes control among adults with type 2 diabetes.

## Introduction

The US Centers for Disease Control and Prevention have reported that an estimated 37.3 million adults (11.3% of the adult population) in the US have type 2 diabetes (T2D).^1^ According to the most recent American Diabetes Association (ADA) lifestyle management guidelines, individuals with T2D should perform at least 150 minutes of moderate-to-vigorous intensity aerobic exercise per week, spread over at least 3 days, and 2 to 3 sessions of resistance training weekly.^2^ Despite these ADA recommendations, approximately 38% of adults with T2D are considered inactive (less than 10 minutes of weekly physical activity), and only 24% meet the current physical activity recommendations.^3^

The value of exercise for glycemic control has been well-established in highly controlled research settings.^4,5,6,7,8,9^ Previous randomized clinical trials have shown the positive effect of combined aerobic exercise and resistance training on glycemic control in T2D.^10,11^ For example, Church et al^10^ demonstrated that thrice-weekly structured exercise sessions including aerobic and resistance training improved hemoglobin A_1c _(HbA_1c_) levels compared with the nonexercise group (−0.34%; 95% CI, −0.64% to −0.03%; *P* = .03), which was not achieved by aerobic exercise or resistance exercise training alone.

Despite the known benefits of regular physical activity for patients with T2D,^10,11,12,13,14^ physician-delivered physical activity advice alone was not effective in increasing physical activity.^15^ In previous studies^10,11,16,17^ of individuals with T2D, exercise adherence rates were high (80%) in highly structured research settings, but adherence to recommended physical activity (46%)^16^ and follow-up attendance rates observed in general population settings were low (50%).^18,19^

In the Initiate and Maintain Physical Activity in Communities Trial (IMPACT) study, we evaluated the effectiveness of a structured exercise program either thrice weekly or once weekly compared with usual care (UC), which consisted of advice only and not taking part in 6-month exercise intervention, in improving HbA_1c_ levels. All participants were instructed to follow the ADA lifestyle management guidelines, with at least 150 minutes of moderate-to-vigorous intensity aerobic exercise per week, spread over at least 3 days, and 2 to 3 sessions of resistance training weekly that encompasses all major muscle groups. We also evaluated whether self-reported physical activity, measured by the Modifiable Activity Questionnaire, and quality of life improved within the 3 groups.

## Methods

### Participants and Screening

Participants for the IMPACT study were recruited from the greater San Francisco, California, Bay Area from 2015 to 2019. A complete rationale and methods have been published elsewhere.^20^ Participants were screened and then randomized after completion of informed written consent and approval by their primary care physician. The Stanford University institutional review board approved this research protocol (Supplement 1). The study follows the Consolidated Standards of Reporting Trials (CONSORT) reporting guidelines for randomized clinical trials.^21^ The study was monitored annually by a data and safety monitoring board.

### Eligibility Criteria

**Figure 1.**
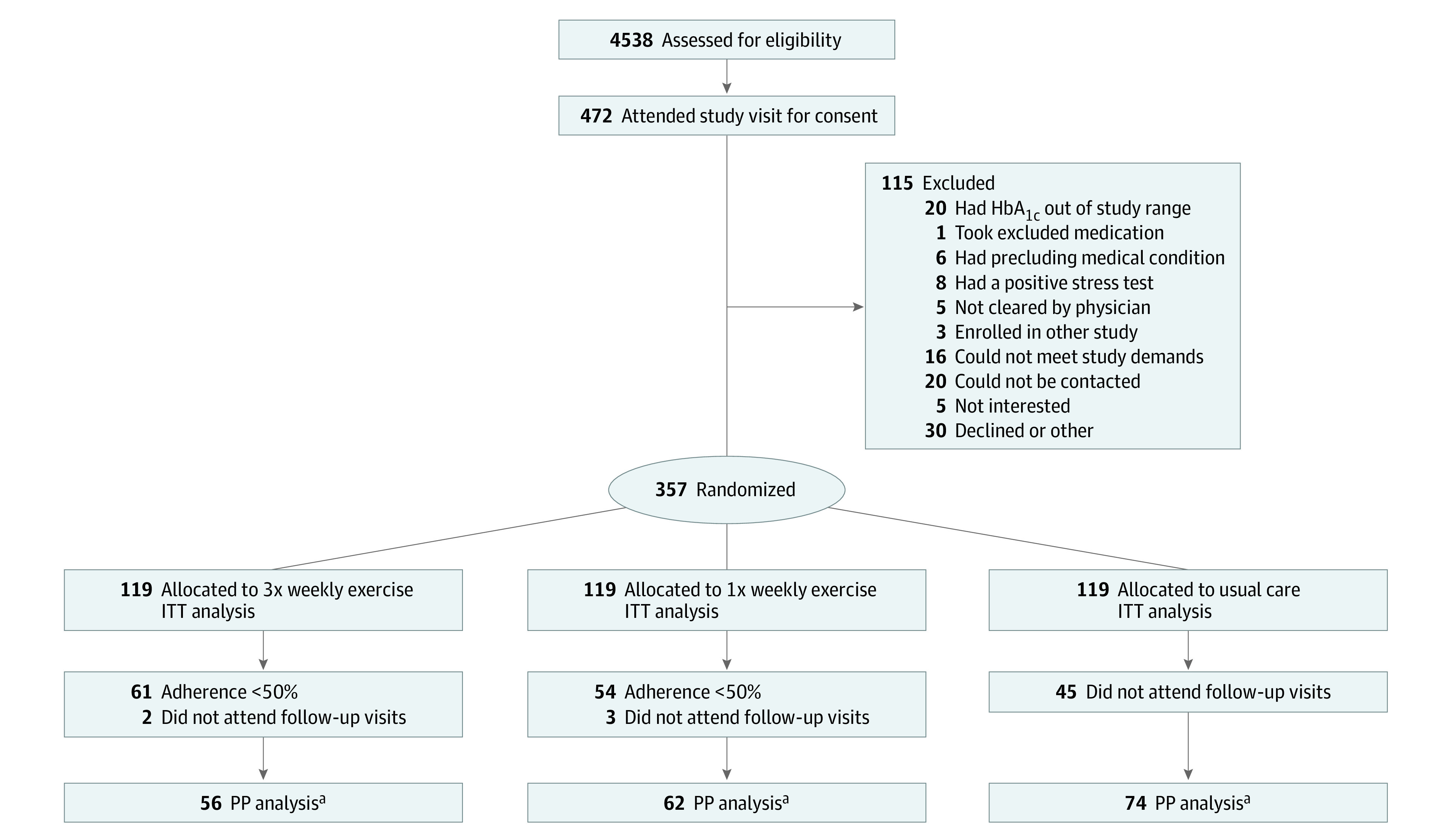
Study Enrollment Flowchart HbA_1c_ indicates hemoglobin A_1c_; ITT, intention-to-treat; PP, per-protocol. ^a^PP is defined as participants who attended at least 11 of 22 expected sessions in the once-weekly exercise group or at least 33 of 66 expected sessions in the thrice-weekly exercise group and attended at least 1 follow-up visit at 3 or 6 months.

Individuals with T2D not taking insulin were randomized (Figure 1). The primary inclusion criteria were age 18 to 80 years (inclusive), diagnosis of T2D without the use of insulin, and HbA_1c_ level of 6.5% to 13.0% (to convert HbA_1c_ to proportion of total hemoglobin, multiply by 0.01). Notable exclusion criteria included body mass index (BMI; calculated as weight in kilograms divided by height in meters squared) greater than 70, HbA_1c_ greater than 13.0%, and any serious medical condition that would contraindicate long-term participation. Race and ethnicity were self-identified by the participants and were assessed in this study to facilitate generalizability assessment and to determine whether intervention effectiveness varied by these factors.

### Study Design

The IMPACT study was a controlled, assessor-blinded, randomized clinical trial using a parallel-group study design with 3 study groups: (1) structured exercise 3 times per week, (2) structured exercise once per week, and (3) UC. Randomization was stratified by baseline HbA_1c_ (low, 6.5%-7.9%; high, 8.0%-9.9%) and age (young, 18.0-49.9 years; old, 50.0-80.0 years) using a block design procedure. All participants were instructed to follow ADA exercise guidelines^22^ and to continue to seek UC from their physicians and were invited to attend monthly group meetings to receive information on healthy lifestyles in diabetes. The exercise groups received similar exercise protocols except for offered session frequency.

### Exercise Interventions

The structured exercise sessions were conducted at more than 60 community-based fitness centers (eg, 24 Hour Fitness, YMCA) in the greater San Francisco Bay Area, covering a geographic range of more than 50 miles. Exercise training staff conducted exercise evaluations every 2 months to adjust exercise recommendations. Participants in the once-weekly exercise group were instructed to attend 1 structured combination exercise session per week (26 sessions total), whereas participants in the thrice-weekly exercise group were instructed to attend 2 structured combination exercise sessions and 1 aerobic exercise session per week (78 sessions total). The combination exercise sessions consisted of a brief warm-up on a treadmill or cycle ergometer (3-5 minutes), aerobic exercise, and resistance training followed by a cooldown. The aerobic exercise consisted of 30 minutes of bicycling, aerobic dancing, walking, and/or jogging. The resistance training involved completing 1 set of 8 different exercises at 8 to 12 repetitions: biceps curl, triceps curl, shoulder press, standing row, abdominal crunch, heel raise, chest press, and squat. Participants were provided an online REDCap portal to report their daily exercise activities, which consisted of attendance at the gym (7212 of 8686 exercise sessions [83%]), independent exercise (at home or while traveling) (634 of 8686 exercise sessions [7%]), or both gym and independent exercise on the same day (840 of 8686 exercise sessions [10%]). Physical activity was assessed by group session attendance, exercise resistance, exercise intensity, and exercise frequency data collected from exercise logs, surveys, and evaluations.

### Outcome Measurements and Follow-up

The primary outcome of the IMPACT study was the absolute change in HbA_1c_ at 3 and 6 months across the 3 groups. HbA_1c_ was analyzed using a DCA Vantage Analyzer (Siemens Healthineers). Antidiabetes medication type and frequency were recorded at baseline and follow-up visits, and the changes in medications were classified as increased, decreased, or unchanged by 2 physicians independently. If a discrepancy was noted between the physicians, a third clinician was consulted, and classification was made by consensus.

Secondary outcomes included changes in self-reported physical activity and self-reported measures of quality of life from baseline to 6 months. Self-reported levels of overall physical activity, including physical activity associated with the intervention, were evaluated with the Modifiable Activity Questionnaire as minutes of metabolic equivalent tasks (MET-minutes) per week.^20,23^ Quality of life was evaluated by the General Health domain from the Short Form Health Survey–12 Item, converted from a 5-point scale to a scale of 0 to 100, with higher scores indicating better general health.^24^ Instances of self-reported physical activity exceeding 3000 MET-minutes per week (3 times US Department of Health and Human Services recommendations)^25^ were excluded as outliers (80 of 692 records [11.6%]). Baseline and follow-up visits were conducted at the University Research clinic site in Palo Alto, California.

### Statistical Analysis

Sample size was determined using data from a previous exercise intervention among individuals with T2D, which showed an effect size of 0.5% reduction in HbA_1c_.^10^ Assuming equal variances, a baseline mean HbA_1c_ of 6.5%, a Bonferroni adjusted α = .05/3 = .017, and power of 80%, we calculated that 92 patients per group would be required to detect a 0.5% difference in HbA_1c_ using a 2-sample *t* test. To account for 20% attrition, we recruited 115 participants per group, resulting in a total sample size of 345.

Primary analysis followed the intention-to-treat (ITT) principle and included all participants according to their randomized intervention. We also performed sensitivity analyses in a per-protocol (PP) cohort limited to all participants who had at least 50% adherence to the structured exercise sessions for the 6-month study period and attended at least 1 follow-up visit (3 or 6 months). Continuous baseline characteristics were presented as median and 25th (Q1) and 75th (Q3) percentiles. Categorical baseline characteristics were expressed as counts and percentages. We computed absolute standardized differences to compare the baseline characteristics by study group assignment.^26^ Between-group differences for baseline characteristics were assessed by protocol adherence using a χ^2^ test or Fisher exact test (for small cell counts) for categorical variables and a *t* test (for normal distribution values) or Mann-Whitney *U* test (for nonnormal distribution values) for continuous measures. We assessed primary and secondary outcomes adjusting for randomization strata (HbA_1c_ and age) with a mean model structure using a constrained longitudinal analysis approach (ie, assumed no difference between study groups at baseline).^27^ The model included a random effect to account for correlation between repeated measures within a participant and assumed an exchangeable correlation structure. We used *P* < .05 (2-tailed) to determine whether there was any significant difference among the 3 study groups at month 6, and if significance was found, we performed pairwise comparisons using a Bonferroni adjusted α = .05/3 = .017 to determine significance between groups.

Data analysis was performed from January to April 2022. All analyses were performed using SAS statistical software version 9.4 (SAS Institute) and R statistical software version 4.0.4 (R Project for Statistical Computing).^28^

## Results

Table 1 shows baseline characteristics of the 357 participants (143 women [40.1%]; mean [SD] age, 57.4 [11.1] years) who were randomized into 1 of 3 study groups: (1) thrice-weekly exercise intervention (119 participants), (2) once-weekly exercise intervention (119 participants), or (3) UC (119 participants). Stratified block randomization yielded balance across groups, as indicated by an absolute standardized difference of less than 0.34 for all baseline demographic variables (Table 1). The median (IQR) HbA_1c_ was 7.4% (6.9%-8.2%), the median BMI (IQR) was 31.3 (28.3-37.2), and most participants (237 participants [66.4%]) had a bachelor’s degree or higher. There were no significant differences in age, sex, or weight between the participants who were and were not at least 50% adherent to exercise in the thrice-weekly exercise group (eTable 1 in Supplement 2).

**Table 1.** Baseline Participant Characteristics

Characteristic	Participants, No. (%) (N = 357)		
	Thrice-weekly exercise (n = 119)	Once-weekly exercise (n = 119)	Usual care (n = 119)
Age, median (IQR), y	59.3 (49.7-65.8)	58.1 (50.5-65.2)	55.8 (48.7-64.9)
Sex			
Female	53 (44.5)	46 (38.7)	44 (37.0)
Male	66 (55.5)	73 (61.3)	75 (63.0)
Race and ethnicity^a^			
African American or Black	10 (8.5)	11 (9.3)	12 (10.2)
Asian	38 (32.2)	50 (42.4)	42 (35.6)
Hispanic or Latino	17 (14.4)	17 (14.4)	16 (13.6)
Non-Hispanic White	42 (35.6)	28 (23.7)	42 (35.6)
Other race^b^	11 (9.3)	12 (10.2)	6 (5.1)
Education^a^			
High school, general educational development, or less	2 (1.7)	10 (8.4)	7 (5.9)
Some college or associate degree	28 (23.5)	38 (31.9)	34 (28.8)
Bachelor’s degree	42 (35.3)	37 (31.1)	28 (23.7)
Graduate degree	47 (39.5)	34 (28.6)	49 (41.5)
Alcohol use^a^			
Never	26 (22.2)	28 (23.9)	28 (24.1)
Rarely	48 (41.0)	40 (34.2)	41 (35.3)
Once or less per week	29 (24.8)	37 (31.6)	23 (19.8)
Twice or more per week	14 (12.0)	12 (10.3)	24 (20.7)
Tobacco use^a^			
Never	92 (77.3)	89 (76.1)	77 (67.5)
Former smoker	20 (16.8)	23 (19.7)	27 (23.7)
Current smoker	7 (5.9)	5 (4.3)	10 (8.8)
Clinical measures, median (IQR)^c^			
Hemoglobin A_1c_, % of total hemoglobin	7.2 (6.8-8.2)	7.3 (6.9-8.2)	7.4 (6.9-8.3)
Weight, kg	86.8 (75.4-106.0)	92.1 (82.0-108.1)	96.0 (84.7-111.6)
Body mass index^d^	30.9 (28.0-35.3)	30.8 (28.6-37.2)	32.8 (28.4-38.3)
Waist circumference, cm	109.0 (100.5-120.0)	110.0 (103.0-122.0)	113.5 (105.0-126.0)
Blood pressure (sitting), mm Hg			
Systolic	129 (119-138)	132 (120-143)	131 (120-141)
Diastolic	84 (78-90)	86 (79-93)	86 (79-92)
Pulse (sitting), beats per minute	76 (70-85)	77 (69-83)	75 (69-85)

SI conversion factor: To convert hemoglobin A_1c_ to proportion of total hemoglobin, multiply by 0.01.

^a^ Race and ethnicity was missing or unknown for 3 participants, education level was missing for 1 participant, alcohol use was missing or declined to answer for 7 participants, and tobacco use was missing or declined to answer for 7 participants.

^b^ Other refers to American Indian/Alaska Native, Pacific Islander, or multiracial.

^c^ Weight was missing for 2 participants, body mass index was missing for 2 participants, waist circumference was missing for 2 participants, systolic blood pressure was missing for 2 participants, diastolic blood pressure was missing for 2 participants, and pulse was missing for 2 participants.

^d^ Body mass index is calculated as weight in kilograms divided by height in meters squared.

Figure 1 provides a detailed diagram regarding patient recruitment, enrollment, and follow-up. From October 2016 to April 2019, a total of 357 individuals with T2D not taking insulin were randomized into the study. Of those randomized, 251 participants (70.3%) completed at least 1 follow-up visit and 192 (54.0%) were included in the PP analysis (Figure 1).

### Effect of Intervention on HbA_1c_

**Figure 2.**
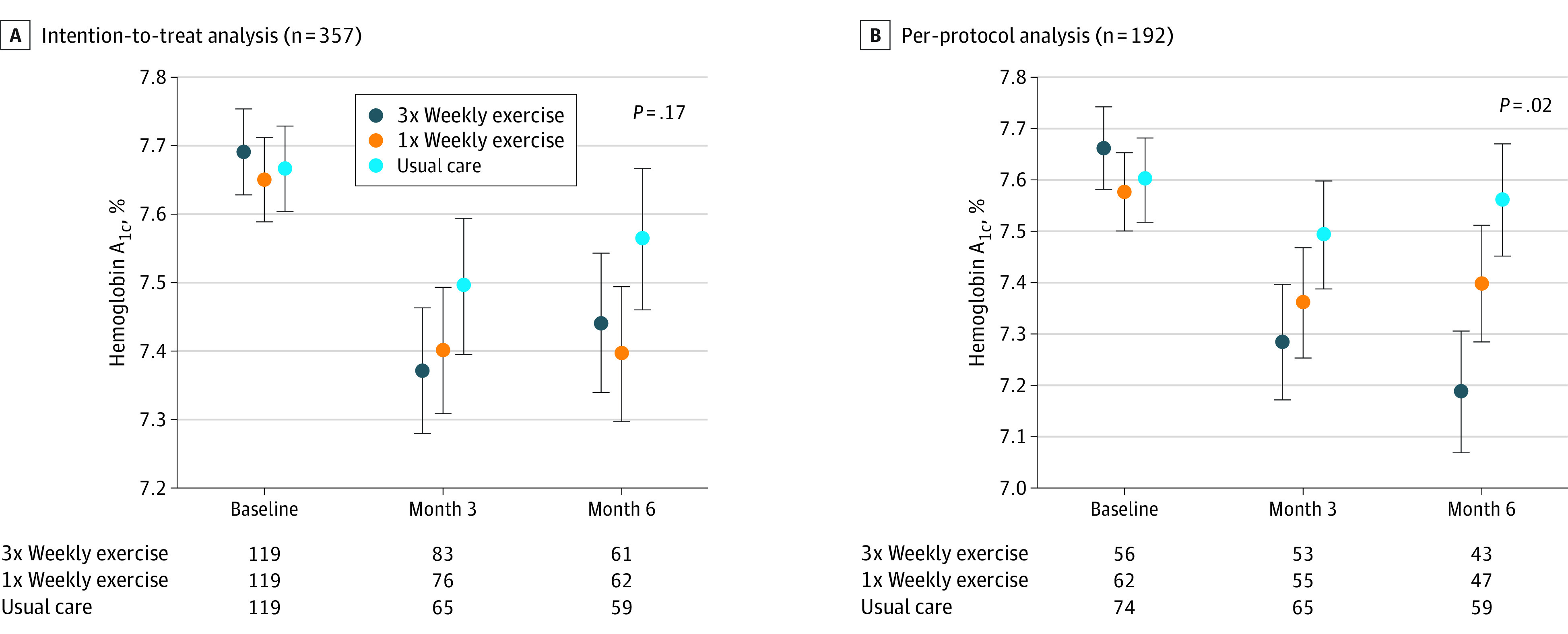
Hemoglobin A_1c _(HbA_1c_) Estimates by Study Group Graphs show the estimated mean HbA_1c_ from the fitted model (Table 2) for each study group at each time point (grouped by time point) along with SEs (denoted by error bars) around each point estimate.

Figure 2 depicts HbA_1c_ levels measured over 3 time points (baseline, 3-month, and 6-month follow-up) across the study groups for the ITT and PP analyses. There was no significant difference in HbA_1c_ among study groups at 3-month or 6-month follow-up in the ITT analysis, as shown in Table 2. Specifically, HbA_1c_ changed by −0.26% (95% CI, –0.48% to −0.03%) in the thrice-weekly exercise group and by −0.18% (95% CI, −0.41% to 0.05%) in the once-weekly exercise group compared with UC at 3 months, and by −0.23% (95% CI, −0.48% to 0.01%) in the thrice-weekly exercise group and −0.16% (95% CI, −0.41% to 0.09%) in the once-weekly exercise group compared with UC at 6 months (*P* > .017 Bonferroni adjusted α for all HbA_1c_ pairwise comparisons).

**Table 2.** Baseline, 3-Month Follow-up, and 6-Month Follow-up Changes From Mixed-Effects Model

Outcome and study group	Intention-to-treat		Per-protocol	
	Adjusted mean (95% CI)	*P* value	Adjusted mean (95% CI)	*P* value
Hemoglobin A_1c_, % of total hemoglobin				
Baseline				
Thrice-weekly exercise	7.69 (7.57 to 7.81)	NA	7.66 (7.50 to 7.82)	NA
Once-weekly exercise	7.65 (7.53 to 7.77)		7.58 (7.43 to 7.73)	
UC	7.67 (7.54 to 7.79)		7.60 (7.44 to 7.76)	
3 mo				
Thrice-weekly exercise	7.37 (7.19 to 7.55)	.08^a^	7.28 (7.06 to 7.51)	.02^a^
Once-weekly exercise	7.40 (7.22 to 7.58)		7.36 (7.15 to 7.57)	
UC	7.49 (7.30 to 7.69)		7.49 (7.28 to 7.70)	
6 mo				
Thrice-weekly exercise	7.44 (7.24 to 7.64)	.17^a^	7.19 (6.95 to 7.42)	.02^a^
Once-weekly exercise	7.40 (7.20 to 7.59)		7.40 (7.17 to 7.62)	
UC	7.56 (7.36 to 7.77)		7.56 (7.35 to 7.78)	
Pairwise comparisons, estimate (95% CI)				
3 mo				
Thrice-weekly exercise vs UC	−0.26 (−0.48 to −0.03)	NA^b^	−0.35 (−0.60 to −0.10)	.005^b^
Once-weekly exercise vs UC	−0.18 (−0.41 to 0.05)		−0.21 (−0.46 to 0.03)	.09^b^
Thrice-weekly exercise vs once-weekly exercise	−0.07 (−0.29 to 0.14)		−0.14 (−0.39 to 0.11)	.28^b^
6 mo				
Thrice-weekly exercise vs UC	−0.23 (−0.48 to 0.01)	NA^b^	−0.38 (−0.65 to −0.12)	.005^b^
Once-weekly exercise vs UC	−0.16 (−0.41 to 0.09)		−0.14 (−0.39 to 0.12)	.30^b^
Thrice-weekly exercise vs once-weekly exercise	−0.07 (−0.32 to 0.17)		−0.24 (−0.52 to 0.03)	.09^b^
Modifiable Activity Questionnaire score				
Baseline				
Thrice-weekly exercise	738 (560 to 916)	NA	735 (513 to 957)	NA
Once-weekly exercise	733 (557 to 908)		730 (530 to 930)	
UC	726 (494 to 958)		704 (471 to 937)	
3 mo				
Thrice-weekly exercise	1126 (855 to 1397)	.005^a^	1277 (1008 to 1546)	.003^a^
Once-weekly exercise	782 (563 to 1002)		866 (624 to 1109)	
UC	831 (536 to 1125)		811 (488 to 1135)	
6 mo				
Thrice-weekly exercise	1108 (799 to 1417)	.01^a^	1269 (926 to 1612)	<.001^a^
Once-weekly exercise	949 (692 to 1206)		983 (721 to 1246)	
UC	656 (209 to 1103)		648 (202 to 1094)	
Pairwise comparisons, estimate (95% CI)				
3 mo				
Thrice-weekly exercise vs UC	304 (63 to 545)	.01^b^	448 (173 to 723)	.001^b^
Once-weekly exercise vs UC	−63 (−310 to 183)	.61^b^	38 (−235 to 310)	.79^b^
Thrice-weekly exercise vs once-weekly exercise	367 (129 to 605)	.003^b^	410 (121 to 700)	.006^b^
6 mo				
Thrice-weekly exercise vs UC	461 (160 to 761)	.003^b^	634 (312 to 957)	<.001^b^
Once-weekly exercise vs UC	274 (−28 to 576)	.08^b^	315 (4 to 626)	.04^b^
Thrice-weekly exercise vs once-weekly exercise	187 (−112 to 486)	.22^b^	319 (−20 to 658)	.07^b^
Short Form Health Survey–12 Item, General Health domain score				
Baseline				
Thrice-weekly exercise	47 (44 to 50)	NA	50 (45 to 55)	NA
Once-weekly exercise	47 (44 to 50)		50 (46 to 55)	
UC	47 (44 to 50)		50 (45 to 54)	
3 mo				
Thrice-weekly exercise	57 (52 to 61)	.15^a^	61 (55 to 67)	.07^a^
Once-weekly exercise	53 (48 to 57)		56 (50 to 62)	
UC	52 (47 to 57)		53 (47 to 59)	
6 mo				
Thrice-weekly exercise	57 (52 to 62)	.25^a^	62 (56 to 69)	.05^a^
Once-weekly exercise	60 (55 to 65)		64 (58 to 70)	
UC	54 (49 to 59)		56 (50 to 62)	
Pairwise comparisons, estimate (95% CI)				
3 mo				
Thrice-weekly exercise vs UC	4.8 (−0.7 to 10.2)	NA^b^	7.1 (0.9 to 13.4)	NA^b^
Once-weekly exercise vs UC	0.4 (−5.1 to 6.0)		1.8 (−4.3 to 7.9)	
Thrice-weekly exercise vs once-weekly exercise	4.3 (−0.9 to 9.6)		5.3 (−1.1 to 11.8)	
6 mo				
Thrice-weekly exercise vs UC	2.5 (−3.5 to 8.4)	NA^b^	5.7 (−0.9 to 12.4)	NA^b^
Once-weekly exercise vs UC	5.1 (−0.9 to 11.1)		7.7 (1.2 to 14.2)	
Thrice-weekly exercise vs once-weekly exercise	−2.6 (−8.4 to 3.2)		−1.9 (−8.8 to 4.9)	

Abbreviations: NA, not applicable; UC, usual care.

SI conversion factor: To convert hemoglobin A_1c_ to proportion of total hemoglobin, multiply by 0.01.

^a^ The overall *P* values correspond to a test of whether there is any difference between the interventions at each time point. The test is performed at a significance level of .05.

^b^ The *P* value corresponds to a pairwise test evaluating whether there are differences between the 2 groups. The test is performed at a significance level of .017 (.05/3 per Bonferroni). The tests are only shown if the overall *P* value for the corresponding time point were <.05.

In the PP subset (62 participants [52.1%] in the once-weekly exercise group and 56 participants [47.1%] in the thrice-weekly exercise group), there were significant differences between study groups at 3-month and 6-month follow-up. Specifically, HbA_1c_ in the thrice-weekly exercise group changed by −0.35% (95% CI, −0.60% to −0.10%; *P* = .005) at 3 months and by −0.38% (95% CI, −0.65% to −0.12%; *P* = .005) at 6 months compared with UC (*P* < .017 Bonferroni adjusted α for both comparisons). HbA_1c_ in the once-weekly exercise group changed by −0.14% (95% CI, −0.39% to 0.12%) compared with UC at 6 months, although the difference was not significant. There were no significant differences observed in thrice-weekly exercise vs the once-weekly exercise study groups. Detailed statistical output is shown in Table 2.

### Adherence and Follow-up

Only 123 of 238 participants (51.7%) in the exercise groups reached at least 50% adherence to exercise sessions. Median overall adherence was comparable in the once-weekly exercise group (54.5%) and thrice-weekly exercise group (47.0%) in the ITT analysis. There were no significant differences in the distance from the university research clinic site between those who were 50% adherent to structured exercise sessions (offered at >60 sites) and those who were not (mean [SD], 14.8 [9.6] miles vs 15.0 [11.3] miles). However, those who did not complete a 6-month follow-up visit lived further away from the university research clinic site (mean [SD], 13.0 [9.0] miles vs 16.7 [12.1] miles; Wilcoxon 2-sample test statistic = 33362.5; *P* = .002). At 6 months, 175 of 357 participants (49.0%) were lost to follow-up.

### Hypoglycemic Medications

Dosage of hypoglycemic medication remained unchanged from baseline to 6-month follow-up for almost 90% of the participants who completed their 6-month study visit. In the thrice-weekly exercise group, 6 participants (5.7%) reduced their dose, 94 participants (88.7%) had unchanged doses, and 6 participants (5.7%) increased their dose. In the once-weekly exercise group, 6 participants (5.8%) reduced their dose, 93 participants (89.4%) had unchanged doses, and 5 participants (4.8%) increased their dose. In the UC group, 3 participants (3.1%) reduced their dose, 87 participants (88.8%) had an unchanged dose, and 8 participants (8.2%) increased their dose. There was no significant difference in medication changes across the 3 groups.

### Effect of Intervention on Self-reported Physical Activity

Table 2 shows significant differences in MET-minutes per week between study groups at both the 3-month and the 6-month follow-up for both the ITT and PP analysis. At 6 months, participants in the thrice-weekly exercise group had 461 (ITT) and 634 (PP) more MET-minutes per week compared with those in the UC group.

eTable 2 and eTable 3 in Supplement 2 show baseline, follow-up, and changes in clinical values in the ITT and PP populations, respectively. eTable 4 in Supplement 2 summarizes the exercise training data by month for individuals who met the per-protocol criteria. Table 2 shows no significant differences in general health in the ITT or PP analysis in any of the study groups.

## Discussion

The main finding of this randomized clinical trial is that a structured exercise program offering exercise thrice or once weekly did not significantly reduce HbA_1c_ in adults with T2D compared with UC. Furthermore, overall adherence to the structured exercise program was low in both exercise groups. There was evidence of HbA_1c_ reduction in the exercise groups, although the effects in the ITT and once-weekly exercise PP analyses did not achieve prespecified levels of significance compared with UC. The thrice-weekly exercise intervention was effective in improving HbA_1c_ in individuals reaching at least 50% adherence to the structured exercise sessions (PP analysis). The once-weekly exercise intervention was not associated with a significant difference from UC in HbA_1c_ levels in the PP analysis, suggesting the importance of offering a higher number of weekly structured exercise sessions to meet ADA exercise recommendations and reduce HbA_1c_.

Participants in the thrice-weekly exercise group with at least 50% adherence had a mean change in HbA_1c_ level (−0.38%; 95% CI, −0.65% to −0.12%) similar to that in a previous study (−0.27%; 95% CI, −0.46% to −0.08%).^10^ These results suggest that a thrice-weekly structured exercise with close monitoring and support of adherence is necessary to make meaningful clinical changes in diabetes severity. These results support offering structured exercise sessions to meet the ADA exercise guidelines for individuals with T2D,^2^ rather than advice alone, as also evidenced in the Activity Counseling Trial.^15^

Compared with UC, participants randomized to the thrice-weekly exercise group (the frequency recommended by ADA) had a substantial increase in their self-reported physical activity at 6 months, whereas no significant difference was observed in the once-weekly exercise group. Furthermore, there was no significant difference observed in the General Health score among the 3 study groups. Many previous studies investigating exercise in individuals with T2D have found exercise has positive effects on general well-being^22^; however, these studies used noncontrolled designs and had a small number of participants enrolled.^29^ Findings in IMPACT were consistent with those of the Diabetes Aerobic and Resistance Exercise study,^29^ whereas the Health Benefits of Aerobic and Resistance Training in Individuals With Type 2 Diabetes study^30^ found that participants in exercise groups did improve their self-reported general health. Additionally, we did not observe improvement in participants’ weight. This is consistent with other trials showing minimal weight loss in exercise-alone interventions, with the greatest weight loss observed in combined diet and exercise interventions.^31,32^ Thus, structured exercise is most beneficial in combination with nutritional recommendations for individuals with T2D for lowering weight and HbA_1c_.

Individuals with T2D are among the least likely to engage in regular physical activity, with participation rates significantly below national norms (58% of US adults are physically active compared with 39% of adults with diabetes).^33^ In addition, their adherence to physical activity programs is as low as 50%.^16,34^ In IMPACT, attrition was similar across study groups. There was a substantial degree of loss to follow-up at 6 months (49%), which is typically observed in general population behavioral interventions (12%-50%).^19,35^ This may be because follow-up was conducted in only 1 clinic site, and we observed that participants who lived further away from this site were less likely to attend a follow-up visit. Regarding adherence, since the IMPACT study did not have a run-in period as an exclusion criterion, we were able to investigate the issue of adherence in a community-based program setting: only 51.7% of participants in the exercise groups reached at least 50% adherence to exercise sessions, similar to other empirical studies. Despite the lower time commitment and greater flexibility to complete exercise on their own in the once-weekly exercise, the median overall adherence was comparable in the once-weekly exercise group (54.5%) and thrice-weekly exercise group (47.0%) in the ITT analysis. This highlights the importance of providing sufficient weekly structured exercise sessions to fulfill the ADA guidelines, rather than fewer structured exercise sessions and recommendations for independent exercise. It is well-established that quitting motivation and confidence are associated with greater adherence to nicotine replacement therapy^36^; however, self-efficacy questions assessing IMPACT participants’ confidence in the intervention and belief that the intervention would be effective were ultimately not associated with exercise adherence or a reduction in HbA_1c_. In the thrice-weekly exercise group, there were no significant differences in demographic characteristics between those who were 50% exercise adherent vs nonadherent. Adherence to exercise sessions at a recommended frequency and duration in a general population setting is a key factor in improving glycemic control.

In the future, we may consider placing follow-up centers physically closer to participants or using mobile data collection. Previous research has identified strategies to improve adherence, including incentives,^37^ on-site study staff monitoring, and on-site fitness coaching,^38,39,40,41^ and using psychological interventions.^42^ Structural, psychological, and social support are critical to ensure adequate adherence to exercise interventions.^43,44^

### Strengths and Limitations

We studied a large sample that had diverse demographic representation using a controlled, randomized study design in a general population setting, making our findings generalizable. We reported change in self-reported physical activity levels in addition to glycemic control.

A limitation of our study is that only 70.3% of participants returned for at least 1 follow-up visit; however, this is consistent with other studies assessing the effect of exercise on individuals with T2D.^16^ This may be attributed to the long distance from many participants’ residences to our clinic site because participants were sampled from a large metropolitan area (approximately 18 000 km^2^), across 135 zip codes, and randomized from 117 zip codes.

## Conclusions

In this randomized clinical trial, there was no significant difference in HbA_1c_ in the ITT analysis. Those at least 50% adherent to the thrice-weekly structured exercise program had a significant reduction in HbA_1c_. Providing thrice-weekly structured exercise is more beneficial than providing once-weekly structured exercise or physical activity advice alone (UC). Future efforts in increasing exercise in T2D should focus on offering structured exercise at least thrice weekly, with additional support to achieve at least 50% adherence to structured exercise programs.

## Notes

Supplement 1.
Trial Protocol and Statistical Analysis Plan

Supplement 2. **eTable 1.** Comparison of Baseline Participant Characteristics: 50% Exercise Adherence vs Nonadherence by Study Group (Per-Protocol Population) **eTable 2.** Baseline, Follow-up, and Changes in Clinical Values in the ITT Population **eTable 3.** Baseline, Follow-up, and Changes in Clinical Values in the PP Population **eTable 4.** Exercise Intervention Data for Individuals Who Met the Per-Protocol Criteria (N = 192)

Supplement 3.
Data Sharing Statement
