# Supplementary material for: Effectiveness of a Community-Based Structured Physical Activity Program for Adults With Type 2 Diabetes: A Randomized Clinical Trial
Source: JAMA Netw Open. 2022 Dec 21;5(12):e2247858. doi: 10.1001/jamanetworkopen.2022.47858 (PMC9857601; doi:10.1001/jamanetworkopen.2022.47858)
Supplement: Supplement 1. — Trial Protocol and Statistical Analysis Plan [file jamanetwopen-e2247858-s001.pdf]

IMPACT Study: Initiate and Maintain Physical Activity in Clinics
Research Protocol

Stanford MEDICINE

TABLE OF CONTENTS

**Chapter 1: Introduction to the IMPACT Research Study**	**4**
1.1 Introduction	5
1.1.a Summary	5
1.1.b Background	5
1.2 Study Research Strategy	5
1.2.a Study Significance	5
1.2.b Study Innovation	8
**Chapter 2: Overview of the Study**	**9**
2.1 Study Purpose and Goal	10
2.1.a Study Purpose	10
2.1.b Study Goal	10
2.1.c Study Intervention	10
2.1.d Study Aims	10
2.2 Study Design	11
2.2.a Randomized Controlled Trial Study	11
2.2.b Longitudinal Study	11
2.2.c Cohort Study	12
2.3 Study Hypothesis	13
2.3.a Primary Hypothesis	13
2.3.b Secondary Hypotheses	13
2.4 Study Population	14
2.4.a Study Population	14
2.4.b Potential Participants	15
2.4.c Inclusion and Exclusion Criteria	16
2.4d Participant Specification	16
**Chapter 3: Participant Recruitment and Enrollment**	**18**
3.1 Participant Recruitment	19
3.1.a Primary Recruitment Methods	19
3.1.b Secondary Recruitment Methods	19
3.1.c Telephone Screenings	20
3.2 Participant Enrollment	21
3.2.a Participant Enrollment	21
3.2.b Baseline Visit	21
3.2.c Notification to Primary Care Providers	24
**Chapter 4: Study Measurements and Intervention**	**25**
4.1 Study Measurements	26
4.1.a Schedule of Study	26
4.1.b Study Visit Measurements	26
4.1.c Exercise Intervention Measurements	28
4.2 Study Intervention	28
4.2.a The IMPACT Exercise Program	28
**Chapter 5: Participant Incentive and Retention**	**33**
5.1 Participant Incentive and Participant Retention	34

5.1.a Participant Incentive 34
5.1.b Participant Retention 34
5.2 Participant Discontinuation 34
5.2.a Participant Discontinuation 34
5.2.b Contingency Plan for Participant Well-Being 35
Chapter 6: Randomization and Data Analysis Plan 36
6.1 Randomization 37
6.1.a Randomization 37
6.2 Data Analytical Methods 38
6.2.a Data Analysis Plan 38
6.2.b Power and Sample Size 41
Chapter 7: Resources and Risk Protection 43
7.1 Study Resources 44
7.1.a IMPACT Study Team 44
7.1.b IMPACT Study Site 44
7.2 Protection Against Risk 44
7.2.a Staff Training 44
7.2.b Protection of Study Information 45
Chapter 8: Data and Safety Monitoring Plan, Adverse Events, and Study Discontinuation 47
8.1 Data and Safety Monitoring Plan 48
8.1.a Data and Safety Monitoring Board 48
8.2 Reporting of Recruitment, Adherence, and Adverse Events 49
8.2.a Recruitment, Adherence, and Compliance 49
8.2.b Adverse Events 49
8.3 Study Discontinuation 52
8.3.a Early Discontinuation of Treatment 52
8.3.b Stopping Rules 52
8.4 Adherence to Ethical, Regulatory, and Administrative Considerations 53
8.4.a Ethical Considerations 53
8.4.b Regulatory Considerations 53
Chapter 9: Data Collection and Management 55
9.1 Data Collection 56
9.1.a Primary and Secondary Data Sources 56
9.2 Data Management 56
9.2.a REDCap Database 56
9.2.b Confidentiality and Protection of Personal Health Information 56
9.2.c Quality Control 57
References 58

Chapter 1: Introduction to the IMPACT Research Study

1.1 INTRODUCTION

1.1.a SUMMARY

The Initiate and Maintain Physical Activity in Clinics (IMPACT) Study is a research study utilizing physical activity in an intervention for Type 2 diabetes (T2DM) patients in a clinical setting. The study will compare structured group exercise within the clinic for T2DM patients compared to usual care, which does not involve structured group exercise within the clinic setting. The main purpose of the study is to determine the optimal and feasible level of frequency of structured contact needed in a clinical setting for adult T2DM patients to initiate and maintain physical activity recommendations long-term.

1.1.b BACKGROUND

Type 2 diabetes (T2DM) affects 25.8 million people in the United States and the prevalence and incidence is increasing.[1] Research has shown that physical activity is beneficial in T2DM management with lower glycemic control achieved when patients follow a structured exercise program in a research setting. Given the known benefits of dietary changes in T2DM, nutrition referrals are common in current clinical practice. Despite the similarly known benefits of exercise,[2-6] physical activity referrals are not readily available in the clinical setting. According to the current American Diabetes Association (ADA) guidelines, individuals with T2DM should engage in at least 150 minutes of moderate-intensity aerobic activity per week and resistance exercises at least 2 times per week.[8] However, approximately 38 percent of T2DM patients do not exercise at recommended levels and 31 percent do not exercise at all.

A recent meta-analysis of randomized controlled trials (RCTs) demonstrated that highly structured physical activity training regimens were effective in reducing glycosylated hemoglobin A1c (HbA1c) levels in individuals with T2DM, while physical activity advice alone had no effect on HbA1c.[2] Of the 23 RCTs which tested structured training programs, most consisted of at least three structured sessions per week, and ranged in length from 3 – 12 months. While the efficacy of such intensive physical activity interventions among individuals with T2DM in a highly structured research setting has been proven, practical approaches to translate and extend these findings into the clinical setting are needed. Less intensive approaches, such as physical activity advice delivered by physicians, have been tested in clinical settings, and are not effective in increasing physical activity.[9]

1.2 STUDY RESEARCH STRATEGY

1.2.a STUDY SIGNIFICANCE

The efficacy of intensive physical activity interventions, consisting of 3 – 5 structured sessions per week among T2DM individuals in a research setting has been proven.[2] Less intensive interventions, such as physical activity advice only, have been attempted in research[2] and

clinical settings[9] are known to be ineffective. Translational studies are needed to test innovative and practical adaptations of evidence-based interventions, in order to legitimize and integrate physical activity as a part of standard clinical practice.

IMPORTANCE OF DIET AND PHYSICAL ACTIVITY IN T2DM MANAGEMENT

Nutrition Counseling in Clinical Practice

The efficacy of nutrition counseling for T2DM was first recognized in clinical trials[10] and with its effectiveness later proven in translational studies.[7,11] In December 1999, the Institute of Medicine released a report[12] confirming the clinical- and cost-effectiveness of nutrition counseling for T2DM. As a result, nutrition counseling became a covered Medicare benefit, and all major insurers followed this reimbursement practice. In 2002, the American Diabetes Association (ADA) recognized the "complexity of nutrition issues" and recommended that specialized services be offered, over and above routine physician office visit counseling, to provide adequate support. For over a decade, nutrition counseling has been successfully institutionalized within the healthcare environment.[12] At the Palo Alto Medical Foundation (PAMF), nearly half of all newly diagnosed T2DM patients receive referrals for specialized diabetes diet education services.[13]

Limited Physical Activity Resources in Clinical Practice

The efficacy of physical activity in T2DM patients is well known. Structured exercise has been shown to improve glycemic control[3,4,13-16] and reduce the risk of cardiovascular disease.[17,18] While the proven value of aerobic exercise is undisputed,[13] resistance training may be particularly helpful for T2DM patients,[19,20] as improved muscle function and growth may increase muscle glucose storage, alter insulin sensitivity via the expression of the GLUT-4 transporter, and thereby improve glycemic control.[21-23] The combination of both aerobic and resistance training exercise has been found to provide the greatest reduction in glycosylated hemoglobin A1c (HbA1c) levels in T2DM patients, compared to either aerobic or resistance training alone. Therefore, the current evidence-based ADA guidelines recommend that individuals with T2DM perform at least 150 minutes per week of moderate-intensity aerobic physical activity and resistance training at least two times per week.[8]

A recent meta-analysis of 47 randomized controlled trials in T2DM has confirmed that structured exercise lowers HbA1c, and physical activity advice alone is ineffective.[2] Despite these findings, structured exercise programs for patients with T2DM are not currently available in clinical settings, and doctors and patients are left to rely on physical activity advice only.[2]

Furthermore, previous research examining physical activity counseling within the clinical setting has shown that physician counseling for physical activity occurs in less than 30% of ambulatory care visits.[26] The Activity Counseling Trial (ACT), a randomized control trial (RCT) designed to compare the effects of two physical activity counseling interventions with recommended physician advice, demonstrated that physician advice and written educational materials did not increase physical activity in patients, both at 6 and 24 month follow-up, compared to baseline.[9] Meta-analysis has confirmed the lack of efficacy of physical activity advice only.[2]

Cardiac rehabilitation, which includes physician prescribed exercise training, is integral to the comprehensive care of patients with cardiovascular disease[24] and is a covered Medicare benefit.[25] Though physical activity is similarly beneficial for T2DM patients, structured exercise programs have not been developed to assist physicians and patients in a clinical setting.

RATIONALE OF TREATMENT AND SELECTION OF INTENSITY DOSES

The current ADA guidelines recommend that individuals with T2DM perform at least 150 minutes per week of moderate-intensity aerobic physical activity and, in the absence of contraindications, perform resistance training at least two times per week, targeting all major muscle groups.[8] The 2006 ADA guidelines[27] also acknowledge the key role of supervision and contact for resistance training, stating that: “To ensure resistance exercises are performed correctly, maximize health benefits, and minimize the risk of injury, we recommend initial supervision and periodic re-assessments by a qualified exercise specialist, as was done in the clinical trials.” The incidence of injury in high-intensity structured exercise trials such as the Health Benefits of Aerobic and Resistance Training in individuals with T2DM (HART-D) study was extremely low (no serious adverse events occurred during exercise training), given that skilled professionals were on site and available to teach proper technique and supervise participants.[3] Conversely, unsupervised physician advice programs, such as the ACT, have yielded much higher rates of musculoskeletal injury (30% annually).[9] These findings underscore the importance of supervision in preventing injury during exercise. Thus, based on the ADA guidelines and previous studies, the IMPACT study has chosen to implement a structured physical activity program based on the HART-D intervention[3] within the clinical setting. Prevention of injury will likely result in longer term adherence to exercise recommendations, and, ultimately, will result in better clinical outcomes.

There is some variation in the frequency of contact and the length of intervention in studies of physical activity and exercise in T2DM patients. Intensive lifestyle interventions have shown that once a week structured contact, in the form of group meetings and individual counseling sessions on diet and exercise, is effective in lowering HbA1c.[28] However, there are no studies of once-weekly interventions of structured physical activity alone. Studies of structured physical activity interventions have differed in dose (≤150 min/week vs. > 150 min/week), and the level HbA1c change differs by the dose of structured physical activity (see Table 1).[2] Most large, recent, and well-designed studies of structured exercise have used frequencies of three times per week, lasting 3-12 months in duration.[2]

Based on previous studies, the IMPACT study has selected a frequency of one (60 min) versus three (180 min) times per week, and an intervention length of 6 months. Based on previous studies at PAMF and literature review, we believe that a minimum of once-weekly contact is necessary to effect significant behavioral changes. Thrice-weekly contact has been shown to be most effective for structured exercise in a research setting. It is unknown whether similar effect sizes can be achieved in the clinical setting, with real-world patients.

1.2.b STUDY INNOVATION

TRANSLATING CLINCIAL TRIAL RESULTS TO CLINICAL PRACTICE

Based on previous studies, determining the correct intervening dosage is essential for effectiveness of behavioral change. While higher dosage interventions work in a research setting, the effectiveness of the dosage level translated into clinical setting remains unknown. An innovative aspect of the IMPACT study is the integration of cost effectiveness, and the consideration of both clinical and patient-centered outcomes in this analysis. Findings from this study will be used to help other healthcare organizations fully realize the investment already made in physical activity research, by translating efficacious clinical trial interventions into clinics.

UTILIZATION OF ELECTRONIC HEALTH RECORD AND PATIENT PORTAL

The Health Information Technology (HITECH) Act[29] calls for the rapid adoption and implementation of electronic health records (EHRs) by medical providers, hospitals, and ambulatory care institutions throughout the country, with the goal of all providers adopting EHR by 2014. SHC has used the EpicCare EHR system since 2008 and is very experienced in the traditional uses of EHR in patient care. The IMPACT study will use the EHR as a tool to operationalize our intervention.

Chapter 2: Overview of the Study

2.1 STUDY PURPOSE AND GOAL

2.1.a STUDY PURPOSE

Translational studies are needed to improve the uptake and effectiveness of physical activity recommendations among the general clinical T2DM patient population. While most other translational studies have explored adapting physical activity interventions to other community settings, the IMPACT study intends to institutionalize physical activity resources within a clinical setting and integrating it with medical care, as this conveys an implicit endorsement of physical activity by a patient's health care team.

2.1.b STUDY GOAL

The goal of IMPACT is to translate efficacious structured physical activity interventions in the clinical setting. Specifically, the study seeks to formalize physical activity as an essential part of T2DM self-management by institutionalizing it within a healthcare setting and empowering patients through structured group exercise therapy.

2.1.c STUDY INTERVENTION

With the addition of resistance training to the 2006 ADA guidelines, it is especially important to provide supervision and support for exercise, to prevent injury and maximize health benefits. In order to address these needs, IMPACT will implement a three-arm RCT to compare the clinical and cost-effectiveness of adding a specialized physical activity component for T2DM patients in a healthcare setting. The intervention will be fully integrated with and supported by the Electronic Health Record (HER). Eligible patients will be recruited, consented, and randomized to one of three study arms. Details of the study intervention will be discussed in a later section.

2.1.d STUDY AIMS

The specific aims of IMPACT study includes: (1) Clinical Effectiveness, (2) Patient-Centered Outcomes, and (3) Cost-Effectiveness.

Clinical Effectiveness
The IMPACT study is to evaluate clinical effectiveness through comparing outcome measures of the three study arms (Arm 1 vs Arm 2 vs Arm 3). The primary outcome will be improvements in Hemoglobin A1c (HbA1c) levels in study participants. Process outcome measures will include changes in physical appearance (ie. body weight, waist circumference), physical fitness (ie. blood pressure, VO_2 max, grip strength), and self-reported physical activity level.

Patient-Centered Outcomes
The IMPACT study is to evaluate patient-centered outcomes using self-reported questionnaires. Satisfaction with the IMPACT exercise program will be compared between the two

experimental study arms (Arm 1 vs Arm 2). Self-reported quality of life will be compared across all three study arms (Arm 1 vs Arm 2 vs Arm 3).

Cost-Effectiveness
The IMPACT study is to evaluate cost-effectiveness based on clinical outcomes and quality of life. This will involve comparing the average cost and outcomes and determining the incremental cost-effectiveness ratio (ICER) for each study arm (Arm 1 vs Arm 2 vs Arm 3).

2.2 STUDY DESIGN

2.2.a RANDOMIZED CONTROLLED TRIAL STUDY

The IMPACT study will implement a three-arm randomized controlled trial (RCT). Of the three study arms, two will be experimental groups with structured exercise sessions (once-weekly or thrice-weekly) as the designed intervention. The remaining study arm will serve as the control group. The study randomization software will assign each eligible study participant into one of three study arms.

Arm 1: Once-Weekly Group Exercise Session (Experimental Group)
The first arm of the IMPACT study requires study participants to attend one on-site group exercise session per week. Each exercise session consists of aerobic and resistance training. In addition, study participants are to complete assigned surveys, update exercise log, and attend a total of six study visits.

Arm 2: Thrice-Weekly Group Exercise Sessions (Experimental Group)
The second arm of the IMPACT study requires study participants to attend three on-site group exercise sessions per week. Participants are to attend one aerobic only session and two combined, aerobic and resistance, sessions. In addition, study participants are to complete assigned surveys, update exercise log, and attend a total of six study visits.

Arm 3: Usual Care (Control Group)
The third arm of the IMPACT study does not required study participants to attend any group exercise sessions. Study participants are to continue with current T2DM management and seek care from their personal healthcare provider. Study participants within this study arm are expected to attend a total of six study visits.

2.2.b LONGITUDINAL STUDY

IMPACT is a longitudinal study designed to track study participants over a course of 2.5 years. Once study participants have been determined eligible and are officially enrolled into the research study, they will begin study phase 1 for six months. After the six months, study phase 2 will begin and last for two years.

Phase 1: Initiate
The first phase of IMPACT (Phase 1) is to initiate physical activity through group exercise sessions for study participants within experimental groups (Arm 1 and Arm 2). The control group (Arm 3) is expected to attend study visits only.

Phase 1 is to take place over a six-month period during which all participants are to attend three study visits (baseline, 3-month, and 6-month).

Phase 2: Maintain
The second phase of IMPACT (Phase 2) is to follow-up with study participants. Experimental study participants (Arm 1 and Arm 2) are monitored to determine whether physical activity is being maintained. The control group (Arm 3) is expected to attend study visits only.

Phase 2 is to take place over a two-year period during which all participants are to attend three study visits (12-month, 18-month, and 30-month).

Study Design Diagram

		Phase 1: Initiate					Phase 2: Maintain		
**ARM**		**V1**		**V2**		**V3**	**V4**	**V5**	**V6**
**1**	R	O_1	X_1	O_2	X_1	O_3	O_4	O_5	O_6
**2**	R	O_1	X_2	O_2	X_2	O_3	O_4	O_5	O_6
**3**	R	O_1		O_2		O_3	O_4	O_5	O_6
	R: Randomization			O: Observations/Outcome Measures				X: Intervention	

2.2.c COHORT STUDY

IMPACT is a cohort study designed to have multiple participant groups over the course of the study. With an anticipated study population of 345 participants.

Each group is expected to commence 15 days apart. The time-period between each cohort group is designed to allow participants in the intervention arms to attend orientation sessions before they start their respective exercise regimen.

2.3 STUDY HYPOTHESES

2.3.a PRIMARY HYPOTHESIS

The primary hypothesis for the IMPACT Study is that structured physical activity interventions in a clinical setting will be efficacious in improving T2DM control, improving physical fitness and increasing physical activity compared to usual T2DM care.

2.3.b SECONDARY HYPOTHESES

Due to the depth and intricacy of the research topic and purpose, IMPACT proposes multiple secondary hypotheses for the study. The hypotheses are categorized according to the specific study aims.

Clinical Effectiveness

1. By the end of Phase 1, experimental study participants (Arm 1 & Arm 2) who adhered to their group exercise schedule should have greater improvements in primary outcomes compared to non-experimental study participants (Arm 3).
    a. Improvements in primary outcomes include reduction in HbA1c levels, increase in VO_2 max levels, and increase in self-reported physical activity levels.

2. By the end of Phase 1, experimental study participants who attended thrice-weekly group exercise sessions (Arm 2) should have greater improvements in primary outcomes compared to experimental study participants who attended once-weekly group exercise sessions (Arm 1).

3. By the end of Phase 2, experimental study participants (Arm 1 & Arm 2) who maintained their exercise schedule should have improved primary outcomes compared to non-experimental study participants (Arm 3).

4. By the end of Phase 2, experimental study participants who maintained thrice-weekly exercise sessions (Arm 2) should have greater improvements in primary outcomes compared to experimental study participants who maintained once-weekly exercise sessions (Arm 1).

Patient-Centered Outcomes

1. By the end of Phase 1, study participants who attended thrice-weekly group exercise sessions (Arm 2) should report greater level of satisfaction with the IMPACT exercise program compared to study participants who attended once-weekly group exercise sessions (Arm 1).

2. By the end of Phase 1, study participants who attended thrice-weekly group exercise sessions (Arm 2) should report greater improvement in quality of life compared to study participants who attended once-weekly group exercise sessions (Arm 1).

3. By the end of Phase 2, study participants who maintained thrice-weekly exercise sessions (Arm 2) should report greater improvement in quality of life compared to study participants who maintained once-weekly exercise sessions (Arm 1).

Cost-Effectiveness

1. Based on the percentage of reduced HbA1c levels, the experimental interventions (Arm 1 & Arm 2) should be more cost-effective compared to the non-experimental intervention (Arm 3).

2. Based on the percentage of reduced HbA1c levels, the once-weekly group exercise intervention (Arm 1) should be more cost-effective compared to the thrice-weekly group exercise intervention (Arm 2).

3. Based on quality of life, the once-weekly group exercise intervention (Arm 1) should be more cost-effective compared to the thrice-weekly group exercise intervention (Arm 2).

2.4 STUDY POPULATION

2.4.a STUDY POPULATION

The population for the IMPACT study consists of individuals who seek care at Stanford Hospitals and Clinics (SHC) and are diagnosed with T2DM.

2.4.b POTENTIAL PARTICIPANTS

Potential study participants for the IMPACT study are patients within the SHC healthcare system. IMPACT will work with the Stanford Center for Clinical Informatics (SCCI) to generate a list of potential participants. Using the EpicCare EHR, the SCCI will query for SHC patients according to the initial screening criteria:

- Alive as of 2016
- Between 30 to 65 years of age
- At least one visit to the SHC in the past two years
- Type 2 diabetic as defined by one or more of the following:
    - Diagnosis of Type 2 Diabetes mellitus (ICD-9 code: 250.X0, 250.X2)
    - Type 2 Diabetes mellitus defined as any two abnormal laboratory values:
        - Hemoglobin A1c results ≥ 6.5%
        - Fasting blood glucose results ≥ 126 mg/dL
        - Random blood glucose ≥ 200 mg/dL
        - Oral glucose tolerance test ≥ 200 mg/dL
    - Current continuous use of any oral anti-diabetic medication
- Recent Hemoglobin A1c (HbA1c) results between 6.6-9.9%

The SCCI will exclude SHC patients with:

- Long-term current use of insulin (ICD-9 code: V58.67)
- BMI > 70 kg/m^2
- Clinical diagnosis of atrial fibrillation (ICD-9 code: 427.3)
- Current pregnancy (ICD-9 code: V22.X)
- Serious concurrent illnesses likely to cause death within the next 5 years (ie. Terminal cancer or obstructive airway disease)

The resulting potential participant list will include the following SHC patient information:

- First Name
- Last Name
- Date of Birth
- Mailing Address
- Contact Telephone Number
- Email address
- Name of Primary Care Provider
- Health Insurance Status
- Preferred Language
- Date of Last HbA1c Value

2.4.c INCLUSION AND EXCLUSION CRITERIA

The purpose of establishing inclusion and exclusion criteria is to define the participant population to be used for the research study. In order for a potential participant to become an eligible participant in the IMPACT study, he or she must meet all of the final eligibility criteria:

- Has primary care physician at SHC
- Diagnosed with Type 2 diabetes
- HbA1c between 6.6 and 9.9%
- Between 30 to 65 years of age
- Has ability to communicate with study staff, sign informed consent, and accept randomization
- Free of intervening events (ie. a sick spouse)
- Willing to exercise at Cardiac Therapy Foundation (CTF) up to three times a week for six months
- Willing to attend all study visits

The IMPACT study has also defined criteria that would exclude an individual from becoming eligible for the study. The criteria for study exclusion are:

- Insulin-dependent
- Resting heart rate ≥120 beats per minute (bpm)
- Blood pressure ≥180/100 mm Hg
- History of or present heart or cardiovascular problems
- History of or present respiratory disease
- History of or present spinal cord injury
- History of stroke or Transient Ischemic Attack (TIA)
- History of cancer diagnosis in the past 5 years or present cancer diagnosis
- Medical, psychiatric, behavioral limitations that may interfere with study participation
- Participating in other clinical trials that may interfere with study procedures and outcomes
- Currently pregnant or plans to become pregnant within three years
- Plans to be away more than four weeks in the next nine months
- Plans to leave the community within five years

2.4.d PARTICIPANT SPECIFICATION

The individuals within the study population have a different designation as they move along the initial processes of recruitment. These designations include: (1) SHC Patients, (2) Potential Participants, and (3) Study Participants.

SHC Patients
SHC Patients are individuals who are a part of the Stanford Healthcare system.

Potential Participants

Potential Participants are SHC Patients who meet the initial screening criteria. These individuals remain as "Potential Participants" throughout the recruitment and baseline visit processes.

Study Participants

Study Participants are Potential Participants who meet the final eligibility criteria. These individuals become "Study Participants" after completing and meeting all criteria during the baseline visit.

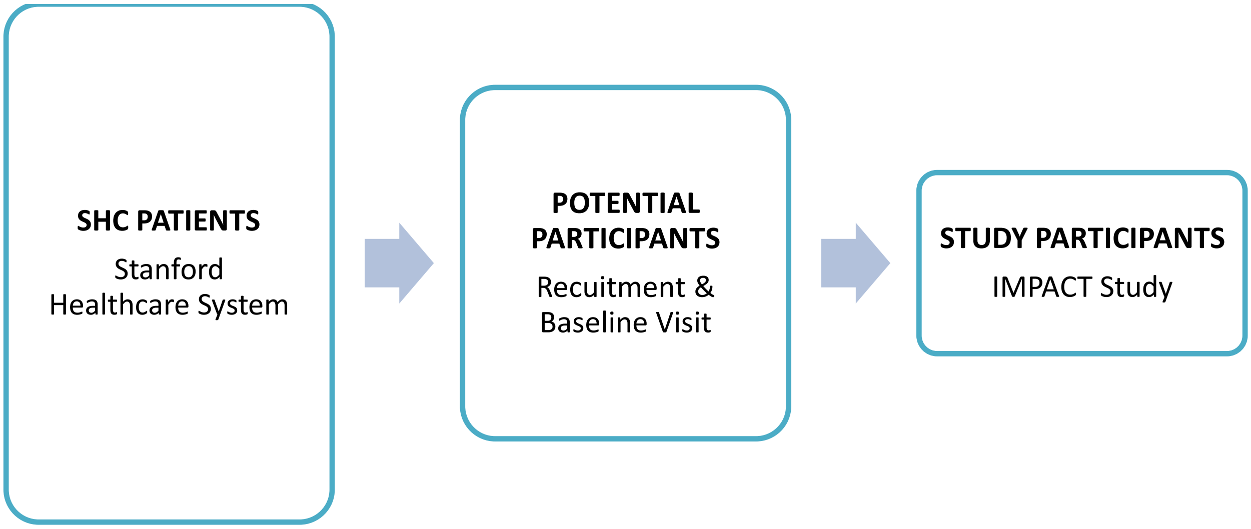

Chapter 3: Participant Recruitment and Enrollment

3.1 PARTICIPANT RECRUITMENT

3.1.a PRIMARY RECRUITMENT METHODS

Primary recruitment methods for the IMPACT study are designed specifically for potential participants. Postal mail will be used for this method. The aim is to provide study information and generate interest from individuals who are likely eligible for the IMPACT study.

POSTAL MAIL RECRUITMENT LETTERS

SCCI will query patient population data and generate a list of study-eligible patients, based on inclusion/exclusion criteria. Once a patient has been identified as a prospective study participant, the PI / study team will obtain approval from the primary care provider (PCP) for contacting his or her patient.

Upon PCP approval, a letter will be sent to potential participant's mailing address. This letter of invitation will explain the purpose of the study and will invite interested participants to contact the PI /study team. In addition to the letter of invitation, potential participants will also receive a return-addressed postcard, allowing potential participants to indicate their interest or disinterest in the research study. Study staff will not contact participants who reply to the postcard and indicate their disinterest in the study and in being contacted.

3.1.b SECONDARY RECRUITMENT METHODS

Secondary recruitment methods for the IMPACT study are designed for the general population. The aim is to raise awareness and identify potential participants through alternate avenues. Depending on the response rate from the primary targeted recruitment methods, secondary recruitment methods may be implemented. Secondary methods to be used for the study include a study webpage, social media, primary care provider, and flyers and brochures.

STANFORD WEBPAGE

IMPACT staff will create a website based on Stanford domain (www.stanford.edu) providing information, methods, objectives of the IMPACT study. This webpage will be updated periodically to provide information on the developments of the IMPACT study.

SOCIAL MEDIA

Facebook and Twitter are the social media recruitment components for the study. The study may utilize social media outlets to announce the commencement and developments of the study. Each social media outlet will include the IMPACT web link to direct readers to information about the study.

PRIMARY CARE PROVIDERS

IMPACT study staff may hold informational meetings with primary care providers and medical assistants to enlist their assistance in referring appropriate patients for the study.

FLYERS AND BROCHURES

Flyers and brochures containing IMPACT study information may be posted and placed in the clinics and labs waiting areas likely to capture the attention of type 2 diabetes mellitus (T2DM) patients (e.g., internal medicine, family medicine, endocrinology). In order to post flyers and place brochures at specific departments, IMPACT staff members will meet with and gain the approval of the relevant department directors.

3.1.c TELEPHONE SCREENINGS

The main purpose of the telephone screening is to inform potential participants of the IMPACT study and determine whether they meet inclusion and exclusion criteria and are study-eligible participants. The telephone screen consists of:

- Greetings and introduction
- Purpose of telephone call
- Brief overview of IMPACT study
- Study description (*when patient is interested in learning more about IMPACT*)
- Screening questions (*when patient is interested in participating in IMPACT*)
- In-person baseline visit scheduling (*when patient is deemed eligible for the baseline visit*)

Telephone screenings are conducted with potential participants who:

- Indicated their interest through direct contact by responding via postal mail and/or telephoning the IMPACT study

If the individual meets the preliminary eligibility criteria based on the screening questions, he or she may continue the study eligibility process. The potential participant will be invited to schedule and attend a study baseline visit to continue the eligibility assessment process. The potential participant is then asked to attend the study baseline visit to begin the assessment process to determine eligibility in study participation.

If the individual does not meet the preliminary criteria based on the screening questions, he or she will not be eligible to participate in the study. If a potential study participant completed an online eligibility questionnaire and did not meet the preliminary criteria, IMPACT study staff will contact that individual to inform them of their ineligibility. The recruitment list will be updated so that participants in the “Do Not Call” list will not be further contacted by IMPACT.

3.2 PARTICIPANT ENROLLMENT

3.2.a PARTICIPANT ENROLLMENT

It is during the baseline visit that the final determination will be made on whether a potential participant is eligible to participate in the IMPACT study. The potential participant must go

through a series of processes before IMPACT staff members can officially enroll him or her into the study.

3.2.b BASELINE VISIT

The baseline visit will consist of multiple steps and must follow a certain order. A potential participant must complete and meet the criteria of each step before continuing onto the next one.

1. Informed consent
2. HbA1c fingerstick test, vital signs measurements
3. Exercise stress test
4. Biobanking
5. Anthropometric measurements
6. MAQ, FFQ, SF-12, EBBS
7. Review of final eligibility checklist
8. Official participant enrollment
9. Randomization
10. Introduction and instruction distribution

Informed Consent

Before anything can take place, informed consent must be obtained from each potential participant. The informed consent process is to provide study information and inform them of their rights as participants. It is also the opportunity for IMPACT staff members to evaluate the competency of the potential participants and their capacity to participate in the study. The informed consent form must be signed in order for the potential participant to continue onto the next process of the baseline visit.

The informed consent form covers the following topics:

- Subject's bill of rights
- Purpose of research
- Duration of study involvement
- Study procedures
- Subject responsibilities
- Study withdrawal
- Possible risks, discomforts, and inconveniences
- Potential benefits
- Subject rights
- Confidentiality
- Financial considerations
- Compensation for research-related injury
- Contact information
- Authorization to use health information for research purposes

During the informed consent process, the staff member is also responsible for evaluating the mental competency of the potential participant. Competency for the IMPACT study is the ability to communicate with staff members and to comprehend the expectations and involvement of the study. Individuals lacking competency will not be invited to participate in the study. Those who are competent and are willing to participate in the study are asked to sign the informed consent form. A staff member will also sign the form. The potential participants are also asked to sign a health information authorization form, which allows IMPACT to utilize their health information for research purposes, and a liability release, which waives any claims against IMPACT and Stanford.

Hemoglobin A1c Fingerstick Test, Vital Signs

Although a potential participant has signed an informed consent form, he or she is not an official study participant. This individual must meet the criteria of the following test and measurements.

Hemoglobin A1c Fingerstick Test

The potential participant must have a baseline hemoglobin A1c (HbA1c) result between 6.6 and 9.9 mg/dL in order to participate. A simple fingerstick test will be performed during the baseline visit. If HbA1c result is out of the desired range, the potential participant is ineligible to participate in the study.

Vital Signs

Blood pressure and resting heart rate are measured to also assess fitness of physical activity. The rationale being that if the blood pressure and/or heart rate for a particular potential participant is already elevated at resting, blood pressure and/or heart rate will elevate to a dangerous level when physical activity is engaged. Therefore, if a potential participant has a resting blood pressure at or above 180/100 mm Hg and/or resting heart rate at or above 120 beats per minute (bpm), he or she is ineligible to participate in the study.

If the above criteria are met, the potential participant is an ideal candidate to participate in IMPACT. He or she must complete the subsequent tests, surveys, and measurements before becoming an official study participant.

Exercise Stress Test

The purpose of the exercise stress test is to measure the effects of exercise on the heart. The results will help the IMPACT study determine whether participation in the exercise program may pose safety concerns for potential participants.

All potential participants must complete an exercise stress test to be eligible for participation. In this event, a potential study participant will be scheduled for an exercise stress test at Stanford Cardiology Lab. This test will take approximately one hour. Potential participants will

be asked to wear comfortable shoes and clothes. Potential participants will monitor their blood glucose on their own glucometer prior to the exercises stress test. The study will provide glucometer if a potential participant forgets to bring their own. The Cardiology Lab will provide the study staff with a research report that identifies eligibility to engage in physical activity. If urgent findings are found, the clinic will notify the Study and the patient's PCP as well.

Anthropometric Measurements

The next process involves anthropometric measurements. IMPACT staff members will take height, weight, and waist circumference measurements for each potential participant.

Modifiable Activity Questionnaire, Block Food Frequency Questionnaire, SF-12 Health Survey, Exercise Barriers and Benefits Scale

The following portion of the baseline visit involves a series of test, surveys, and questionnaires. The potential participant must complete all of the tools in order to proceed.

Modifiable Activity Questionnaire

Modifiable Activity Questionnaire (MAQ) assesses physical activity levels. The potential participant is asked to recall the types, frequency, and duration of engaged exercise activities for the past three months. MAQ also evaluates sedentary levels through its supplemental questions.

Block Food Frequency Questionnaire

The Block Food Frequency Questionnaire (FFQ) assesses dietary intake. The potential participant is asked to recall the types and frequency of specific food item consumed in the past 3 months. The FFQ is administered online through NutritionQuest, the company that developed the tool. IMPACT staff members will set up a NutritionQuest account for each participant.

SF-12 Health Survey

SF-12 Health Survey (SF-12) evaluates quality of life. The potential participant is asked to complete a 12-question survey on his or her self-perceived physical and mental health status.

Exercise Barriers and Benefits Scale

Exercise Barriers and Benefits Scale (EBBS) evaluates the perception of exercising. The responses on the scale are scored to determine if the potential participant has a positive or negative perception on exercise.

Review of Final Eligibility Checklist

Once the potential participant completes and meets the criteria of all the measurements, tests, surveys, and questionnaires mentioned above, the eligibility checklist must undergo review. Some of the screening questions used during the telephone screening will be revisited to ensure the potential participant response has not changed over time.

An IMPACT staff member will make a final review of the checklist and determine whether the potential participant is eligible to participate in the study. If the potential participant is declared eligible, he or she is an official study participant. He or she will complete a demographic information form and undergo the randomization process.

Randomization

Randomization is the process utilized to determine the study arm for each study participant. The specific randomization method to be used by IMPACT will be discussed in a later section. Once the study participant is ready to be randomized, his or her information will be inputted into the randomization software. Based on the provided information, the study participant will be assigned to the once-weekly experimental group (Arm 1), the thrice-weekly experimental group (Arm 2), or the usual care non-experimental group (Arm 3).

Introduction and Instruction Distribution

Once a study participant has been randomized, an IMPACT staff member will introduce and discuss the components of the assigned study arm with the participant. Every study participant will receive an IMPACT Participant Handbook. Study participants in Arm 1 and Arm 2 will also receive an IMPACT Exercise Handbook.

Biobanking

IMPACT staff will help potential participants schedule a blood draw appointment at the Stanford Freidenrich Center for Clinical and Translational Research (CTRU) within three weeks time from the baseline visit. Participants will be asked to fast for 8-10 hours prior to the blood draw. This appointment will take approximately 15 minutes and up to 75 milliliters of blood will be drawn and stored for future research projects. Participants will be asked to provide a urine sample during this visit as well.

3.2.c NOTIFICATION TO PRIMARY CARE PROVIDERS

Following the Baseline Visit, the IMPACT Study will contact the primary care providers (PCPs) of the study participants via an encrypted email. PCPs will be notified of their patients' participation in the IMPACT Study. Additionally, detailed information regarding IMPACT will be provided to PCPs.

Chapter 4: Study Measurements

4.1 STUDY MEASUREMENTS

4.1.a SCHEDULE OF STUDY

The expected duration of the study is approximately 2.5 years for each participant. Phase 1 of the IMPACT study is 6 months in duration while Phase 2 is 24 months in duration. Over the course of the study, study participants will be asked to attend six study visits. Three study visits are to occur during Phase 1 and Phase 2 each. The first visit participants are to attend is their baseline with subsequent visits to occur 3 months, 6 months, 12 months, 18 months, and 30 months after their baseline visit.

	PHASE 1: INITIATE			PHASE 2: MAINTAIN		
MONTH	0	3	6	12	18	30
VISIT	V1	V2	V3	V4	V5	V6

4.1.b STUDY VISIT MEASUREMENTS

Measurements collected for the IMPACT Study will vary depending on the intervention arm and study visits. The following is a list of essential measurements to be collected during the course of the six study visits.

- Demographics
  - Age
  - Gender
  - Race and ethnicity
  - Religion
- Diabetes status
  - Hemoglobin A1c (HbA1c) level
- Anthropometric
  - Height
  - Weight
  - Waist circumference, Hip circumference
- Vital signs
  - Blood pressure
  - Heart rate
- Physical activity capacity
  - Exercise stress test
  - VO2 max
- Survey and questionnaires
  - Modifiable Activity Questionnaire (MAQ)
  - Block Food Frequency Questionniare (FFQ)
  - SF-12 Health Survey (SF-12)
  - Exercise Barriers and Benefits Survey (EBBS)

- Patient Satisfaction Questionnaire (PSQ)

- Resource utilization
  - Participant attendance
  - Staff presence

Measurement/ Activity	V1 Baseline	R1	R2	V2 3-Month	V3 6-Month	V4 12-Month	V5 18-Month	V6 30-Month
**Participant Management**								
Informed consent	X							
PCP Clearance	X*							
Final Eligibility Checklist			X					
Randomization			X					
**Clinical and Physical Assessments**								
HbA1c POCT	X			X	X	X	X	X
Blood pressure, heart rate	X			X	X	X	X	X
Height, weight, Waist:Hip Circumference		X		X	X	X	X	X
**Forms, Surveys, Questionnaires**								
Demographic Form	X							
SF-12 Health Survey	X			X	X	X	X	X
Modifiable Activity Questionnaire		X		X	X	X	X	X
Exercise Benefits and Barriers Scale		X			X			X
Food Frequency Questionnaire			X	X	X	X	X	X
Patient Satisfaction Questionnaire				X	X			
**Assessments Not Performed by IMPACT Study Team**								
Exercise Stress Test	X**							
Biobank		X***						

* PCP clearance will be obtained prior to recruitment screening and at a second point prior to enrollment in the study.
** Participants will be directed to undergo a stress test with Stanford Cardiology Clinic after their baseline visit is complete and prior to the completion of R2.
*** Participants will be scheduled appointments at CTRU within three weeks from the Baseline visit for blood draw and urine sample.

4.1.c EXERCISE INTERVENTION MEASUREMENTS

Additional measurements will be collected from study participants specifically in the exercise intervention groups (Arm 1 and Arm 2). The following is a list of essential measurements to be collected over the course of the six-month exercise intervention period.

- Blood glucose control
  - Daily blood glucose levels
- Physical fitness
  - Strength
  - Exercise intensity
- Physical activity level
  - Activity type
  - Activity duration
  - Activity intensity
  - Activity location
- Resource utilization
  - Participant attendance
  - Staff presence

Cross-Sectional Exercise Intervention Measurements

Measurements	Month #1	Month #2	Month #3	Month #4	Month #5	Month #6
Physical Fitness	X	X		X		

Continuous Exercise Intervention Measurements

Measurements	Month #1	Month #2	Month #3	Month #4	Month #5	Month #6
Blood Glucose Control	→	→	→	→	→	→
Physical Activity Level	→	→	→	→	→	→
Resource Utilization	→	→	→	→	→	→

4.2 STUDY INTERVENTION

4.2.a THE IMPACT EXERCISE PROGRAM

The study intervention is an exercise program developed by the IMPACT Study and reviewed by Dr. Joseph Ciccolo, PhD, Dr. Christian Roberts, PhD, and Haideh Plock, DPT, ACT, OCS. Dr. Ciccolo is an expert in exercise physiology and exercise psychology and an Assistant Professor at Brown University. Dr. Roberts is an expert in physiological science and an Associate Research Professor at UCLA School of Nursing. Ms. Plock has extensive expertise in physical therapy and is currently the Manager at the PAMF Physical Therapy Center.

The IMPACT Exercise Program is designed for study participants in Arm 1 and Arm 2 to engage in structured exercise activities in a clinical setting. The structured exercise sessions are led by instructors and conducted in groups. The entire exercise program is 26 weeks or approximately six months in length and will take place during Phase 1 of the study.

Once study participants are assigned to a study arm, they have entered Study Phase 1. Study participants in both Arm 1 and Arm 2 must schedule and undergo an initial exercise evaluation before beginning the exercise program.

Exercise Evaluations
Over the course of the intervention period, study participants will attend a total of three exercise evaluation sessions; before the intervention starts, at 8 weeks, and at 16 weeks. The purpose of these evaluations is to assess physical fitness levels and to ensure exercise routines are appropriate for study participants. The initial exercise evaluation will help establish personalized exercise regimens. Based on the results of this evaluation, exercise routines will be personalized according to strength and intensity levels. The second and third exercise evaluations will be used to monitor participant progress and modify routines when necessary.

Group Exercise Sessions
The exercise session consists of aerobic training and resistance training. The estimated duration of each session is 60 minutes. The exercise session begins with warm-up stretches. Aerobic training is to take place first with resistance training to follow after. The session concludes with cool-down stretches.

Aerobic Training Session
The aerobic training session consists of aerobic exercises only. The estimated duration of each session is 40 minutes. The session consists of a warm-up walk and followed by one of the three categories of aerobic training. The session concludes with cool-down activities.

Resistance Training

Resistance training, or strength training, can improve overall health and serves as a complement to aerobic training. Engaging in resistance exercises helps individuals build and maintain muscle. For T2DM patients, resistance exercises can improve insulin sensitivity and reduce blood glucose levels.

WARM-UP (7-8 MINS)

- Active stretches
- Range of motion exercises
- Cardio warm-up exercises

AEROBIC TRAINING (30 MINS)

- Bicycling (stationary or recumbent bike), walking, jogging, dancing

RESISTANCE TRAINING & COOL DOWN (22-23 MINS)

- Bicep curl
- Tricep curl
- Chest Press
- Standing row
- Shoulder press
- Abdomen crunch
- Squats
- Heel raise
- Static stretches

Muscle Group	Arms	Chest	Shoulder	Back	Abdomen	Legs
Exercises	• Bicep curl<br>• Tricep curl	• Chest press	• Shoulder press	• Standing row	• Crunch	• Squat<br>• Heel raise

Repetition Schedule

The repetition recommended for each resistance exercise will vary according to the exercise period. This schedule is based on the ACSM exercise recommendations with revisions by Dr. Joseph Ciccolo, Dr. Christian Roberts, and Dr. Neil Johannsen.

	Exercise Period #1	Exercise Period #2	Exercise Period #3
Number of Sets	1	1	1
Number of Repetitions	10-15	10-15	10-15

The repetition recommended for each exercise may vary for individual study participants. The ranges for number of repetitions allow study participants to exercise at a lower difficulty if needed or higher difficulty when possible. The IMPACT exercise program also includes alternate resistance exercises to accommodate specific exercise intensity needs.

Location

All group exercise sessions will take place at the Cardiac Therapy Foundation (CTF) located at 4000 Middlefield Rd, Palo Alto, CA 94303.

Instructors

Group exercise sessions are led by trained CTF staff members.

Equipment

All equipment required for the group exercise sessions are provided by CTF or the IMPACT study. Equipment includes free weights (dumbbells), cardio machines (bikes) and resistance bands. The IMPACT Study will provide additional equipment, such as glucometers, heart monitors, and fast-acting carbohydrates (snack, juice, glucose tablets).

PERSONAL EXERCISE REGIMEN

Study participants may choose to exercise outside of the IMPACT group exercise sessions. To prevent introducing biases to the study, IMPACT will not encourage or instruct study participants to exercise independently. However, IMPACT will not discourage study participants from personal exercise. The personal exercise regimen refers to exercise activities outside of the structured group exercise sessions, which may occur at home or elsewhere.

During the initial resistance exercise evaluation, each study participant will be provided with an exercise resistance band based on the outcomes of the evaluation session. The level of

resistance of the provided band should closely match the recommended free weights for the study participant. Study participants will be taught to perform the following exercises using the resistance band.

Major Muscle Group	Exercise
Arms (biceps)	Bicep Curl
Arms (triceps)	Tricep Curl
Chest	Chest Press
Shoulder	Shoulder Press
Back	Standing Row

Besides resistance band exercises, study participants are free to engage in any other indoor or outdoor physical activities. Activities include but are not limited to: casual walking, brisk walking, walking stairs, jogging, and biking. Study participants are asked to record their personal exercise activities in their exercise logs.

Chapter 5: Participant Retention and Discontinuation

5.1 PARTICIPANT INCENTIVE AND PARTICIPANT RETENTION

5.1.a PARTICIPANT INCENTIVE

Since the goal of the IMPACT study is to translate structured physical activity in the clinical setting, the study environment will simulate and closely replicate real-world practices. Study participants will not receive any monetary compensation for their participation. However, study participants may receive small non-monetary compensation such as water bottles, and tote bags.

5.1.b PARTICIPANT RETENTION

The IMPACT study plans to employ various methods in order to retain study participation.

Reminders and Scheduling
To ease some of the burden of the study participants, IMPACT staff members will contact study participants via telephone and/or email to remind them of their upcoming study appointments and to schedule their visit and exercise sessions. The schedules for study visits and exercise sessions will have a degree of flexibility to accommodate the study participants.

Wellness Seminars and Newsletters
While IMPACT is not offering monetary incentives, the study aims to offer incentives that promote positive health behaviors. IMPACT will develop and invite healthcare professors to provide wellness seminars specifically for study participants. IMPACT will also create monthly newsletters with health, nutrition, and wellness topics for study participants.

Appreciation Events
The IMPACT Study will hold appreciation events for its participants on an annual basis. These events provide the study participants an opportunity to learn about the progression of the study and also to meet and interact with study staff and other participants.

5.2 PARTICIPANT DISCONTINUATION

5.2.a PARTICIPANT DISCONTINUATION

The IMPACT study is based on voluntary participation. In other words, potential participants and study participants have the right to refuse participation and withdraw from the study at any time.

Circumstances may also arise where the study physician or nurse determines that a participant must withdraw from the study. Circumstances will be reviewed on an individual basis to determine if withdrawal from the study is to be temporary or permanent.

5.2.b CONTIGENCY PLAN FOR PARTICIPANT WELL-BEING

Study participants will be advised to seek medical care or advice from their usual healthcare provider for any conditions that may arise during the course of the study. If a participant is at a study visit when the medical condition occurs, the on-site study physician or study nurse will be notified immediately. IMPACT staff members are trained to recognize common life-threatening situations and can provide Basic Life Support (BLS) when necessary. If the medical condition is non-threatening but requires attention, the participant will be brought to SHC in its Fast Track program via the Emergency Department. If the medical condition is severe and life-threatening, IMPACT staff members will call 9-1-1 for emergency assistance. The IMPACT Study will send secure message via EpicCare to notify participant primary care provider (PCP) any medical occurrences.

Chapter 6: Randomization and Data Analysis Plan

6.1 RANDOMIZATION

6.1.a RANDOMIZATION

Block Randomization is the method to be used to randomize study participants into study arms. The method is developed to pre-specify the number of arm assignments in a random order. IMPACT will utilize Block Randomization in conjunction with stratification in order to promote balance of participant characteristics within each study arm. The participant characteristics, or parameters, to be used for the IMPACT study includes: (1) age, (2) gender, and (3) HbA1c level.

Participant characteristics will be stratified as followed:

- Age (years)
  - >29.9 – 50.9 (young age)
  - 51.0 – 65.0 (older age)
- Gender
  - Male
  - Female
- HbA1c (percentage)
  - 6.6 – 7.9 (low A1c)
  - 8.00 – 9.9 (high A1c)

As a result, there are eight (8) strata in total:

1. Low HbA1c, younger age and female
2. Low HbA1c, younger age and male
3. Low HbA1c, older age and female
4. Low HbA1c, older age and male
5. High HbA1c, younger age and female
6. High HbA1c, younger age and male
7. High HbA1c, older age and female
8. High HbA1c, older age and male

Each block size will contain six (6) group assignments, evenly distributed across the three study arms. The blocks will contain random permutations (ie. 211332, 321231, etc) that are pre-determined by SAS.

Randomization Envelopes
IMPACT Biostatistician will prepare a total of eight (8) folders, one for each stratum. The specific group assignment within the folder will be included in six (6) sealed envelopes with the sequence within block on them.

Baseline Visit
Randomization of study participants will take place during baseline visits. Once an individual has been officially enrolled into the IMPACT study, a staff member will review participant

characteristics and determine corresponding stratum. The participant will be given an envelope containing his or her study group assignment.

6.2 DATA ANALYTICAL METHODS

6.2.a DATA ANALYSIS PLAN

Statistical analyses will investigate clinical effectiveness of structured physical activity regimens across varying frequencies. The main statistical approach will be to date-match outcomes to concomitant exercise regimens, then to examine univariate and multivariate associations with changes in clinical outcomes, patient-centered outcomes, and cost. Statistical significance will be determined at $P<0.01$, a stricter value due to the expected number of models used, or with other adjustments for multiple comparisons when applicable. Statistical analyses will be performed using SAS® (Cary, NC), and supplemented with STATA or Splus for methods not available in SAS®.

Study participants who are not able or willing to continue with the randomized frequency of intervention will be treated as loss to follow-up and only data obtained while in the assigned protocol will be used in the primary analysis (intention-to-treat). Since participants and non-participants continue to receive care within the healthcare system contemporaneously during the research study, IMPACT will obtain interstitial outcome data and complete clinical narrative through observational data available in the EHR. The statistical analysis will be conducted by Senior Statistician/s with the guidance of Biostatistical Consultant Robert Tibshirani, PhD.

DESCRIPTIVE STATISTICS

Descriptive statistics will be used to describe the participant population at baseline. Participant characteristics will include: (1) Age, (2) Gender, (3) Race, (4) Ethnicity, and (5) Education Level, (6) Body Mass Index (BMI), (7) Hemoglobin A1c (HbA1c) Level, (8) Comorbidities (ie. hypertension and dyslipidemia), (9) Dietary Characteristics, (10), Physical Activity, and (11) Pharmacotherapy.

SUB-GROUP ANALYSES

Analyses will be performed within sub-groups to compare the primary outcome of absolute change in percent HbA1c and process measures of changes in VO_2max and self-reported physical activity:

1. Years of age at study enrollment. 30-45 vs. 45-55 vs. 56-65
2. Gender. Male versus female.
3. Race. White vs. African American/Black vs. American Indian vs. Asian vs. Pacific Islander vs. Other
4. Ethnicity. Non-Hispanic/Latino vs. Hispanic/Latino

STUDY AIM 1: CLINICAL EFFECTIVENESS

Research Questions

1. Does a thrice-weekly, structured exercise regimen have greater reduction in HbA1c than a weekly contact exercise regimen? How do these interventions compare to usual care?
2. Are the same improvements observed for process measures (i.e., VO2 max and self-reported physical activity)?

Primary Outcomes and Process Measures

Primary outcomes and process measures will be compared between the three intervention arms (weekly, thrice-weekly, usual care). Primary outcomes include absolute change in percent HbA1c, and process measures include change in VO2 max and self-reported physical activity. Adherence will be defined as attending more than 80% of assigned structured exercise sessions, and will be used as a covariate for stratification or adjustment.

Statistical Analysis

In univariate analyses, the percent change from baseline will be compared across intervention arms using appropriate parametric (ANOVA, *t* tests) or non-parametric (Wilcoxon) methods.

In multivariate analyses, change in outcomes will be modeled using linear regression. Percent change from baseline will be a continuous outcome with intervention arm as the predictor of interest and various participant characteristics as covariates (e.g. sex, age, race/ethnicity, anthropometry, self-reported diet and exercise, pharmacotherapy, family history, etc.). Repeated measures analysis using the outcome at different time points and mixed-effects models will also be performed. Available nonparticipants will serve as contemporaneous comparators to address possible secular trends in outcome values.

Study participants in the exercise program may also be undergoing concomitant pharmaceutical therapy to reduce HbA1c. The approach is to repeat the above analyses stratified by use of hypoglycemic medication. Another approach is to use a composite binary outcome variable for whether participants were successful in either decreasing diabetes medication or reduced HbA1c by 0.5% without increasing medications. Logistic regression will be used to assess the likelihood of achieving the composite outcome.

Study participants will be studied based on their randomized assignment (intention-to-treat). In sensitivity analyses, participants may be re-analyzed based on actualized attendance if they more closely resemble one of the other arms (once-weekly, thrice-weekly, usual care). A study participant assigned to thrice-weekly, who attends only once a week may be re-analyzed as a weekly patient. Stratification by adherent (attendance >80%) or not will also be another sensitivity analysis.

STUDY AIM 2: PATIENT-CENTERED OUTCOMES

Research Questions

1. Does thrice-weekly contact improve patient satisfaction with the program?
2. Does thrice-weekly contact improve quality of life?

Satisfaction and quality of life will be assessed using self-reported questionnaires, Patient Satisfaction Questionnaire (PSQ-30) and SF-12 Health Survey. The two instruments will be individually compared across the two intervention arms (once vs. thrice-weekly) and across all three study arms (once-weekly vs. thrice-weekly vs. usual care).

Categorical answers to the questions will be compared using appropriate chi-squared tests (univariate), and logistic or multinomial models (multivariate). Univariate comparisons of quality of life scale (based on SF-12®) (bounded 0 to 1) across the three arms will be assessed using ANOVA and post-hoc pairwise tests with an inverse normal transformation.

Quality of life will be related to participant characteristics (i.e., age, sex, race/ethnicity, anthropometry, nutrition, pharmacotherapy, family history, exercise in the home environment, etc) to assess which patient factors are associated with increased quality of life. Mixed-effects modeling and clustering methods will be employed. Clustering methods, such as hierarchical clustering, will be used to identify clusters of participant that have similar quality of life levels. Then a comparison of the participant characteristics of those identified in the same cluster will be performed to understand patterns in quality of life scores.

STUDY AIM 3: COST EFFECTIVENESS

Research Questions

1. What is the most cost-effective frequency of PAD-SMAs based on percentage lowering of HbA1c?
2. What is the most cost-effective frequency of PAD-SMA sessions based on quality of life?

The average cost and outcomes of each intervention arm and the usual care arm will be first assessed. Using each cost and effectiveness measure, cost-effectiveness of the three levels of interventions will be computed in comparison to the usual care group.

Cost-effectiveness will be assessed with two indices: (1) average cost-effectiveness for each intervention and control group, defined as $(\text{total costs})_A/(\text{average effectiveness})_A$ for group A, and (2) incremental cost-effectiveness ratio (ICER) of groups A vs. B, defined as $\{(\text{total cost})_A-(\text{total cost})_B\}/\{(\text{effectiveness})_A-(\text{effectiveness})_B\}$. The ICER indicates the expected incremental cost of each intervention arm over no intervention per unit improvement in effectiveness.

Confidence intervals with bootstrapping standard errors will be used to elicit statistical inferences of each index. The indices using varying costing and effectiveness metrics will be compared. By study design, significant differences across the three intervention arms in patient demographic (age, gender, race/ethnicity), clinical (co-morbidities) or behavioral (i.e. adherence) characteristics are not expected. However, if systematic difference in patient characteristics between intervention and usual care group is detected, alternative models specifically adjusting for selection issues will be employed, such as propensity score matching and selection models. Furthermore, heterogeneous treatment effects with subgroup analysis

based on patient gender, age, and baseline clinical conditions (in BMI, HbA1c levels) will be explicitly examined.

STUDY RECRUITMENT ANALYSIS

Analyses related to study recruitment will be performed. Methods of participant recruitment, electronic versus postal mail recruitment, will be summarized and compared. Participant response rates according to the individual recruitment methods will also be determined.

Simple analyses will also be conducted to summarize the amount of effort in participant recruitment. This will include tabulating the number of calls made to potential participants, the number of voice messages left on voicemail boxes, the total amount of time for all phone screens and the average amount of time for a single phone screen. In addition, the frequency and percentage of various phone screening outcomes: (1) Completed, (2) Incomplete, (3) Not Interested, and (4) Lost to Follow-Up, will be examined.

6.2.b POWER AND SAMPLE SIZE

The primary analyses for this study will be comparing the clinical outcomes, patient-centered outcomes, and cost effectiveness of structured exercise regimens across varying frequencies (once vs. thrice-weekly, compared to usual care). For Specific Aim 1, overall change in baseline of outcome values will be examined (e.g. HbA1c, VO_2max, physical activity). By the algebraic properties of log and variance, we estimated difference in groups as $\log(HbA1c_1)-\log(HbA1c_2)=\log(HbA1c_1/HbA1c_2)$, and SD(log(HbA1c))=SD(HbA1c)/mean(HbA1c). A two-sample *t*-test between means, Bonferroni adjusted alpha=0.05/3=0.016, assuming normality of log(HbA1c), equal group variances, and a baseline mean HbA1c of 6.5%, we will have 80% power to detect a 0.5% point difference in HbA1c with 92 patients/arm. Accounting for a 20% attrition, this will require 115/arm, 345 patients total.

Specific Aim 2 seeks to compare the satisfaction of patients between groups as measured by self-reported satisfaction questionnaires. The data will be collected using ordered, multi-point Likert scales, and in some cases responses will be dichotomized (e.g. 5 points with 1-3 vs 4-5). For a power/sample size estimate, we used a difference in proportion estimate. Using a likelihood ratio, chi-squared test of two proportions, alpha=0.05/3=0.016, power=0.80 and an underlying referent satisfaction of 80% and equal sample sizes of 92-115/arm per above, we are able to detect minimum differences in proportions of 15 percentage points, and minimum detectable odds ratios 4.9. To compare the quality of life metrics of patients between groups as measured by SF-12,® the data will be collected using SF-12® and appropriately converted to a bounded value between 0 and 1. In analyses, we will use the inverse normal transformation of quality of life to obtain a normally distributed quality of life value. For a power/sample size estimate, we estimated the difference in score, using a two-sample t test with pool variance of 0.09, the Delta Method and Taylor Series expansion, properties of the inverse normal transformation, alpha=0.05/3=0.016, power=0.80, and equal sample sizes of 92-115/arm per above. We are able to detect a minimum difference in quality of life scores of 0.09 (e.g. 0.50 vs 0.59 would be detectable, on a scale from 0-1).

Cost-effectiveness (Specific Aim 3) analyses use aggregate cost and effectiveness values, along with confidence intervals derived from a bootstrap method. Therefore, sample size meeting the criteria for the above two aims would be sufficient for Specific Aim 3. Missing data will be mitigated through retention strategies (see Section C.8) and augmentation with EHR data (see Section C.5.3). For patients missing some, but not all, follow-up data, the missing data mechanism will be explored and modern imputation methods such as multiple imputations will be used to impute missing covariates following satisfaction of the method's assumptions.

Chapter 7: Resources and Risk Protection

7.1 STUDY RESOURCES

7.1.a IMPACT STUDY TEAM

Dr. Latha Palaniappan (PI) is clinical professor at Stanford University. Dr. Palaniappan and the team of qualified study consultants (Timothy Church, Joseph Ciccolo, Neil Johannsen, Trevor Orchard, and Christian Roberts) are well-versed in clinical trial design and implementation.

Sundar Thapaliya, project coordinator is responsible for the planning and implementation of the IMPACT Study. SCCI and Jiaqi Hu, Biostatistician provide informatics assistance on data collection and data management. Dr. Robert Tibshirani (Biostatistical Consultant) will provide advice on statistical methodology and guide the analyses as appropriate.

Robin Wedell, RN, FPCNA, is the directors of CTF, and will serve as the site PI at Cardiac Therapy Foundation.

Dr. Sukyung Chung (Co-Investigator) is a Research Economist and has methodological expertise in the economic evaluation of health care interventions and is experienced in analyzing EHR data in T2DM patient population.

Additional members of the study team include members from Dr. Palaniappan's Research Team; Katie Hastings, MPH (Lab Manager), Maria Bregendahl, MS (Clinical Research Coordinator), Abbie Reguindin, BS (Social Science Research Coordinator), Nadejda Marques, PhD (Diabetes paraprofessional, Study Staff) .

7.1.b IMPACT STUDY SITE

The IMPACT Study will be conducted at Stanford University and CTF. Study visits will take place at: (1) 1070 Arastradero Road Suite 100 Palo Alto, CA, Assessment Rooms (2) Cardiology Clinic at 300 Pasteur Drive, Stanford 94304 (3) Stanford Freidenrich Center for Clinical and Translational Research (CTRU) at 800 Welch Road Stanford, CA 94304. Group exercise sessions will take place at the CTF at 4000 Middlefield Road, Palo Alto 94303.

7.2 PROTECTION AGAINST RISK

7.2.a STAFF TRAINING

Dr. Latha Palaniappan (PI) will monitor completion of regulatory documentation, including approval of the protocol and Informed Consent forms by the Stanford Institutional Review Board (IRB) and certification that each member of the study team has complied with regulations for Protection of Human Subjects training and HIPAA. The IMPACT Study will not be performed until an appropriate IRB submission and consent form unique to this study has been

approved. All examination procedures pose little risk to participants. However, research staff members are trained to respond to all health emergencies by first contacting the emergency response system (9-1-1), then notifying appropriate medical personnel on-site. In addition, the Principal Investigator will supervise the training of clinic staff in human subjects certification, informed consent and assent procedures, and the collection of data.

7.2.b PROTECTION OF STUDY INFORMATION

CONFIDENTIALITY

To protect study participants, data will be strictly confidential. Only study team members and study-related personnel will have access to participant's private health information / health information identifiers (e.g., first and last name, patient DOB, address and phone number) in order to schedule, run and conduct the study. Stanford University will share participants' identifying information and health information with the Cardiac Therapy Foundation only as necessary to conduct the study and to ensure participant safety.

All participants will be identified by a unique study ID, which will be generated as part of the study enrollment process. Data containing any identifying information such as a name or address will be kept separately from the study ID and other data collected for the study. Only the PI / study team will have access to the key code. No participant data will be stored on local drives or laptops.

Every effort will be made to keep identifying information confidential. Any printed materials containing information on participants (e.g., consent forms) will be stored in locked filing cabinets within locked offices, with access by the PI / study team only. No study participant will be identified individually in any publication. Participant names and identifiers will not be used in the publication or presentation of findings from this study.

Database development work, constructing data from multiple sources including primary data collection and entry, and EpicCare EHR, will be carried out by the SCCI/IMPACT analyst team, Database files will be maintained in a computer that will require passwords for access known only by study personnel. All data from study visits and intervention exercise sessions will be directly entered into the secure study database on a secure, password protected computer.

SECURITY AWARENESS TRAINING

All study team members will receive training on policies regarding the confidential nature of the data collected, processed, and stored at Stanford, and must sign a confidentiality agreement before being allowed access to confidential information. The training will cover topics such as the HIPAA regulations, protection of participant rights, privacy and confidentiality, disposal of confidential material, etc. The Principal Investigator will also reinforce the confidential nature of all study data at study team meetings.

All study team members will receive training and pass requirements for conducting research on human subjects. The Stanford provides education and certification on human subjects research

through the Collaborative IRB Training Initiative (CITI), a web-based training package on issues relating to human subjects research. The CITI web site is maintained by the University of Miami, with content developed by a national consortium. Over 400 institutions use CITI for mandatory Protection of Human Subjects training. Successful completion of the CITI Basic Course is required of all faculty, staff, and students who are engaged in research activities at Stanford that involves human subjects.

PROTECTION AGAINST DATA LOSS

A backup of the study database will be made daily.

MACLICIOUS CODE PROTECTION

Stanford uses several techniques to protect against assaults by viruses, spy bots, worms and other malicious attacks.

Chapter 8: Data and Safety Monitoring Plan, Adverse Events, and Study Discontinuation

8.1 DATA AND SAFETY MONITORING PLAN

8.1.a DATA AND SAFETY MONITORING BOARD

The IMPACT Study will utilize a Data Safety Monitoring Board (DSMB).

OBJECTIVES

The major goals of Data Safety Monitoring Board (DSMB) are to:

1. Review safety data, including but not limited to adverse events, serious adverse events (i.e., hemorrhage, death), and appropriateness of subject enrollment
2. Ensure protection of privacy
3. Ensure confidentiality of data
4. Assure intervention goals are being met

SIZE AND COMPOSITION

The DSMB will consist of 3 to 5 members both internal and external to the IMPACT team and consultants. The planned composition may include the following: Biostatistician (1), Exercise expert (1-2), and Clinician (1-2).

MAJOR RESPONSIBILITIES

1. Sign and abide by a statement of confidentiality
2. Disclose any actual or potential conflicts of interest
3. Familiarity with research protocol and plans for safety monitoring
4. Oversee safety of participants to include review of adverse events
5. Review reports of related studies, as appropriate
6. Review major proposed modifications

Following each meeting, the DSMB will provide written documentation regarding findings for the study as a whole and any relevant recommendations related to continuing, changing, or terminating the study.

APPOINTMENT OF MEMBERS

DSMB voting members are appointed by the Principal Investigator and/or her designee for a fixed term.

Meetings

DSMB meetings will take place annually (in-person) and on a quarterly basis (by teleconference) until there are no active participants in the study.

Recommendations

All DSMB recommendations are submitted to the Principal Investigator and/or her designee with a copy provided to NIH Program Director or designee.

Reports

The frequency of data review for this study differs according to the type of data, the availability of the data collected, and the perceived level of risk.

Data Type	Frequency of Review
Recruitment (adherence to demographics and inclusion/exclusion stated in protocol)	Quarterly
Adverse event rates	Quarterly
Compliance to intervention	Quarterly
Statistical power implications due to dropouts and missing data	Annually
Progression of diabetes, increases in blood pressure, and/or worsening of lipid profiles in the three study groups	Annually

8.2 REPORTING OF RECRUITMENT, ADHERENCE AND ADVERSE EVENTS

8.2.a RECRUITMENT, ADHERENCE AND COMPLIANCE

RECRUITMENT AND ADHERENCE

Review of the rate of recruitment as well as adherence to inclusion or exclusion criteria occurs annually to confirm that participants meet the eligibility criteria as stated in the grant proposal.

PARTICIPANT COMPLIANCE

Interventionists monitor participant compliance to the intervention protocol on a weekly basis. This data is shared with the Principal Investigator, Research Coordinator, Statistician, and the Safety Officer. If the Safety Officer has any concerns about the compliance inhibiting the ability of the study to test the primary outcome, study investigators will be contacted and methods for improving compliance will be discussed.

8.2.b ADVERSE EVENTS

Adverse events will be presented to the Study Principal Investigator, Statistician, Chair of the Stanford University Institutional Review Board. Adverse event data from the intervention groups will be analyzed quarterly. The IMPACT Study anticipates most adverse events will be mild and the participant will be able to resume intervention activities within a day or two of reporting the event.

DEFINING ADVERSE EVENTS

By definition, an adverse event is any unfavorable or unintended sign or symptom temporarily associated with the medical assessment or procedure or the intervention.

Event Classification

Adverse events are categorized based on event outcomes.

1. Serious Adverse Event – Any event that results in the following outcomes: Death, risk of death (life-threatening), in-patient hospitalization or prolongation of hospitalization, a persistent or significant disability or incapacity, or congenital anomaly or birth defect.
2. Unexpected Adverse Event – Any adverse event, the specificity or severity of which is not an expected consequence of the medical treatment or procedure.

Event Attribution

Adverse events may also be defined by attribution. Attribution refers to the determination of whether an adverse event is related to a medical treatment or procedure. The categories for attribution are as followed:

1. Definite – The adverse event *is clearly related* to the medical treatment or procedure.
2. Probable – The adverse event *is likely related* to the medical treatment or procedure.
3. Possible – The adverse event *may be related* to the medical treatment or procedure.
4. Unlikely – The adverse event *is doubtfully related* to the medical treatment or procedure.

Event Severity

For the IMPACT Study, adverse events will be further graded according to severity. Appropriate actions related to participant well-being and study intervention will be taken according to the severity of the event.

Grade 1: Mild Adverse Events

A mild adverse event includes slight muscle soreness or stiffness that does not limit daily activity. Modifications to an intervention are not needed for a Grade 1 adverse event. More frequent evaluations may be required until the adverse event resolves or the study participant stabilizes.

Grade 2: Moderate Adverse Events

Moderate adverse events include severe muscle soreness or stiffness resulting in a limitation in daily activity. Study participants who develop Grade 2 adverse events may continue to exercise at full protocol prescription. However, the intervention may be modified until the adverse event resolves. In addition, more frequent evaluations may be required until the adverse event resolves or the study participant stabilizes.

Grade 3: Severe Adverse Events

Severe adverse events include severe muscle soreness or stiffness that results in limited daily activity. Severe adverse events also include other physical events including, muscle strain, ligament injury, intermittent claudication, development of transient but reproducible dyspnea, chest discomfort, or lightheadedness. The intervention for study participants who develop Grade 3 adverse events will be modified until the adverse event returns to Grade 1 or less. More frequent evaluations may be required until the adverse event resolves or the study participant stabilizes.

If a severe adverse event persists despite a modified intervention, the intervention may be interrupted for up to a maximum of 2 weeks. If the intervention must be interrupted for more than 2 weeks, the participant may be permanently discontinued from the study. If the adverse event resolves within the 2 weeks, the modified intervention may be restarted. A study participant who tolerates the modified intervention for at least 2 weeks can then be increased to the full intervention.

Grade 4: Life-Threatening Adverse Events

Life-threatening adverse events include bone fracture or development of refractory myocardial ischemia, dyspnea, and clinically relevant contraindications for exercise as outlined by the American College of Sports Medicine. Study participants who develop Grade 4 adverse events will have their intervention discontinued permanently. The study participant will return for a follow-up evaluation as clinically indicated or in a maximum of 2 weeks and should remain under medical observation until the adverse event resolves or the study participant is stabilized.

REPORTING SPECIFIC ADVERSE EVENTS

All serious and/or unexpected adverse events regardless of causality will be reported within 48 hours (2 calendar days) of notification of the event to the Chair of the Institutional Review Board, and Chair of the Data Safety Monitoring Board. Deaths or life-threatening events related to study intervention will be reported within 24 hours of notification.

Examples of IMPACT Study-Related Adverse Events
Examples of specific adverse events, which require immediate reporting include, but are not limited to the following:

1. Life-threatening adverse events
2. Inpatient hospitalization
3. Prolongation of an existing hospitalization
4. Disability
5. Occurrence of chest pain
6. Intermittent claudication
7. Cardiac or pulmonary complications as recommended by the American College of Sports Medicine
8. Any death that occurs while the patient is enrolled in the study including the follow-up period, or within 30 days of completing the study.

8.3 STUDY DISCONTINUATION

8.3.a EARLY DISCONTINUATION OF TREATMENT

If a study participant is removed from the study prior to completion of the intervention, the reason and date will be documented. If a study participant is removed from the study, the follow-up assessments will be obtained. If the study participant is removed because of intolerance or complications related to the intervention, the study participant would remain under medical observation until resolution or stabilization of the adverse event. Participants will be removed from the study for any of the following reasons:

- The study participant has a serious or life-threatening adverse event.
- The study participant develops serious illness.
- The study participant becomes pregnant during the study term.
- The study participant begins insulin therapy.
- The study participant enrolls in a clinical trial which interferes with study procedures and outcomes.
- The study participant suffers physical injury preventing the continuation of study intervention.
- The investigator feels that it is in the best interest of the study participant to withdraw.
- The study participant wishes to withdraw.
- The study participant fails to comply with the intervention protocol, assessments, or other requirements of the study.

8.3.b STOPPING RULES

There is minimal risk for participating in this research study. Participation in physical activity may improve risk factors. The most likely scenario that would indicate a cessation of the study would be failure to recruit participants or implement the intervention as planned. However, in

addition to monitoring recruitment and compliance to the intervention, the IMPACT Study also monitors the rates of injury in study participants. The Safety Officer, in conjunction with the study investigators, will alert the National Institute of Health (NIH) and DSMB if a larger than reasonably expected injury rate occurs in the treatment groups. Other issues that are related to the stopping rules include:

1. New information – It is unlikely that new information will become available during this study that would result in discontinuing the trial.
2. Limits of assumption – It is possible that the value of data analysis will be limited by differences between the intervention groups at baseline or because of study drop-outs/or missing data. Baseline differences will be analyzed annually and effects on the power to detect differences in the outcome measures will be evaluated and discussed with the PI, Safety Officer, and the NIH. If the study dropout rate exceeds 15%, the Safety Officer will initiate a meeting with the PI to discuss strategies to increase retention. If the dropout rate exceeds 25%, the safety officer will meet with the study investigators to determine whether or not the study should continue.
3. Limit of rules – The IMPACT Study acknowledges that circumstances, other than what are listed, may justify stopping the study.

8.4 ADHERENCE TO ETHICAL, REGULATORY AND ADMINISTRATIVE CONSIDERATIONS

8.4.a ETHICAL CONSIDERATIONS

The IMPACT Study is responsible for obtaining IRB approval from Stanford University prior to study initiation. Study investigators will be responsible for submitting and forwarding reports to the IRB and DSMB.

INFORMATION FOR PARTICIPANTS

Before obtaining consent, participants will be informed of the objectives, benefits, risk and requirements of the IMPACT Study.

Informed Consent

All participants must provide their informed consent prior to any study visit activities. The written consent form will be the most current version that has been reviewed and approved by IRB. Informed consent will be obtained by the IMPACT study staff and the forms will be signed by the participants and the IMPACT staff who conducted the informed consent discussion. A copy of the signed forms will be made. The participants will have a copy of the form while the study will keep the original form in participant files.

8.4.b REGULATORY CONSIDERATIONS

FINANCING

The IMPACT Study was funded by the National Institute of Diabetes and Digestive and Kidney Diseases (NIDDK) at National Institutes of Health (NIH).

TRIAL REGISTRATION

IMPACT has been registered on ClincialTrials.gov as an efficacy study in February 2014. Its ClincialTrials.gov identifier is NCT02061579.

DISCLOSURE OF CONFLICT OF INTEREST

Full disclosure by all of the key members of the IMPACT Study of their, and their immediate family's financial relationships with organizations just to have an active or potential interest in the conduct and outcome of the study. These are to be reported and reviewed annually.

Chapter 9: Data Collection and Management

9.1 DATA COLLECTION

9.1.a PRIMARY AND SECONDARY DATA SOURCES

8.1.a Primary and Secondary Data Sources

The IMPACT Study utilizes primary data sources for study research and analysis. IMPACT has designed and selected various collection tools to gather data for specific study needs. Secondary data sources are used only if data are missing from primary sources.

Primary Data Sources

Primary data sources are methods and tools utilized by IMPACT to collect information directly from its participants. This includes all participant information collected during or from:

- Telephone/online screenings
- Baseline study visits and subsequent study visits
- Paper and electronic surveys and questionnaires
- Exercise evaluation sessions
- Group exercise sessions
- Participant exercise logs

Secondary Data Sources

Secondary data sources are existing datasets collected by another entity. The IMPACT Study will use EpicCare EHR to obtain HbA1c results and other metabolic measurements if participants do not attend their study visits.

9.2 DATA MANAGEMENT

9.2.a REDCAP DATABASE

REDCap is a web-based application designed to build and manage online databases and surveys. REDCap is widely used at Stanford and has been approved for use on human subjects research studies.

The IMPACT Database is developed by the IMPACT study team. The database consists of a combination of data collection form and survey tools. The IMPACT Study is utilizing REDCap to store and manage study data

Data Entry

Collected study data is either entered directly into REDCap during study visits or entered into REDCap after being collected onto paper forms. The exact method of data entry will be determined according to resource availability and time efficiency.

9.2.b CONFIDENTIALITY AND PROTECTION OF PERSONAL HEALTH INFORMATION

To protect study participants, data will be strictly confidential. Only study team members and study-related personnel will have access to participant's private health information / health

information identifiers (e.g., first and last name, patient DOB, address and phone number) in order to schedule, run and conduct the study. Stanford University will share participants' identifying information and health information with the Cardiac Therapy Foundation only as necessary to conduct the study and to ensure participant safety.

All participants will be identified by a unique study ID, which will be generated as part of the study enrollment process. Data containing any identifying information such as a name or address will be kept separately from the study ID and other data collected for the study. Only the PI / study team will have access to the key code. No participant data will be stored on local drives or laptops.

Every effort will be made to keep identifying information confidential. Any printed materials containing information on participants (e.g., consent forms) will be stored in locked filing cabinets within locked offices, with access by the PI / study team only. No study participant will be identified individually in any publication. Participant names and identifiers will not be used in the publication or presentation of findings from this study.

Database development work, constructing data from multiple sources including primary data collection and entry, and EpicCare EHR, will be carried out by the SCCI/IMPACT analyst team, Database files will be maintained in a computer that will require passwords for access known only by study personnel. All data from study visits and intervention exercise sessions will be directly entered into the secure study database on a secure, password protected computer.

9.2.c QUALITY CONTROL

To ensure the quality of study data, IMPACT staff will be responsible for data checking and editing within a reasonable period of time. The IMPACT Study Coordinator will select and review a random selection of participant files quarterly for data entry accuracy and completeness.

References

REFERENCES

1. Centers for Disease Control and Prevention. National Diabetes Fact Sheet. 2011; http://www.cdc.gov/diabetes/pubs/factsheet11.htm. Accessed September 12, 2011.
2. Umpierre D, Ribeiro PA, Kramer CK, et al. Physical activity advice only or structured exercise training and association with HbA1c levels in type 2 diabetes: a systematic review and meta-analysis. *JAMA.* May 4 2011;305(17):1790-1799.
3. Church TS, Blair SN, Cocreham S, et al. Effects of aerobic and resistance training on hemoglobin A1c levels in patients with type 2 diabetes: a randomized controlled trial. *JAMA.* Nov 24 2010;304(20):2253-2262.
4. Sigal RJ, Kenny GP, Boule NG, et al. Effects of aerobic training, resistance training, or both on glycemic control in type 2 diabetes: a randomized trial. *Ann Intern Med.* Sep 18 2007;147(6):357-369.
5. Balducci S, Leonetti F, Di Mario U, Fallucca F. Is a long-term aerobic plus resistance training program feasible for and effective on metabolic profiles in type 2 diabetic patients? *Diabetes Care.* Mar 2004;27(3):841-842.
6. Lambers S, Van Laethem C, Van Acker K, Calders P. Influence of combined exercise training on indices of obesity, diabetes and cardiovascular risk in type 2 diabetes patients. *Clin Rehabil.* Jun 2008;22(6):483-492.
7. Sadur CN, Moline N, Costa M, et al. Diabetes management in a health maintenance organization. Efficacy of care management using cluster visits. *Diabetes Care.* Dec 1999;22(12):2011-2017.
8. American Diabetes Association. What We Recommend. 2015; http://www.diabetes.org/food-and-fitness/fitness/types-of-activity/what-we-recommend.html
9. The Writing Group for the Activity Counseling Trial Research Group. Effects of physical activity counseling in primary care: the Activity Counseling Trial: a randomized controlled trial. *JAMA.* Aug 8 2001;286(6):677-687.
10. UK Prospective Diabetes Study 7: response of fasting plasma glucose to diet therapy in newly presenting type II diabetic patients, UKPDS Group. *Metabolism.* Sep 1990;39(9):905-912.
11. Franz MJ, Monk A, Barry B, et al. Effectiveness of medical nutrition therapy provided by dietitians in the management of non-insulin-dependent diabetes mellitus: a randomized, controlled clinical trial. *J Am Diet Assoc.* Sep 1995;95(9):1009-1017.
12. Institute of Medicine. *The Role of Nutrition in Maintaining Health in the Nation's Elderly: Evaluating Coverage of Nutrition Services for the Medicare Population*. Washington, DC: National Academy Press; 2000.
13. Boule NG, Haddad E, Kenny GP, Wells GA, Sigal RJ. Effects of exercise on glycemic control and body mass in type 2 diabetes mellitus: a meta-analysis of controlled clinical trials. *JAMA.* Sep 12 2001;286(10):1218-1227.
14. Boule NG, Kenny GP, Haddad E, Wells GA, Sigal RJ. Meta-analysis of the effect of structured exercise training on cardiorespiratory fitness in Type 2 diabetes mellitus. *Diabetologia.* Aug 2003;46(8):1071-1081.
15. Eriksson J, Taimela S, Eriksson K, Parviainen S, Peltonen J, Kujala U. Resistance training in the treatment of non-insulin-dependent diabetes mellitus. *Int J Sports Med.* May 1997;18(4):242-246.

16. Dunstan DW, Daly RM, Owen N, et al. High-intensity resistance training improves glycemic control in older patients with type 2 diabetes. *Diabetes Care.* Oct 2002;25(10):1729-1736.
17. Hu FB, Stampfer MJ, Solomon C, et al. Physical activity and risk for cardiovascular events in diabetic women. *Ann Intern Med.* Jan 16 2001;134(2):96-105.
18. Wei M, Gibbons LW, Kampert JB, Nichaman MZ, Blair SN. Low cardiorespiratory fitness and physical inactivity as predictors of mortality in men with type 2 diabetes. *Ann Intern Med.* Apr 18 2000;132(8):605-611.
19. Tresierras MA, Balady GJ. Resistance training in the treatment of diabetes and obesity: mechanisms and outcomes. *J Cardiopulm Rehabil Prev.* Mar-Apr 2009;29(2):67-75.
20. Gordon BA, Benson AC, Bird SR, Fraser SF. Resistance training improves metabolic health in type 2 diabetes: a systematic review. *Diabetes Res Clin Pract.* Feb 2009;83(2):157-175.
21. Baldi JC, Snowling N. Resistance training improves glycaemic control in obese type 2 diabetic men. *Int J Sports Med.* Aug 2003;24(6):419-423.
22. Castaneda C, Layne JE, Munoz-Orians L, et al. A randomized controlled trial of resistance exercise training to improve glycemic control in older adults with type 2 diabetes. *Diabetes Care.* Dec 2002;25(12):2335-2341.
23. Richter EA, Jensen P, Kiens B, Kristiansen S. Sarcolemmal glucose transport and GLUT-4 translocation during exercise are diminished by endurance training. *Am J Physiol.* Jan 1998;274(1 Pt 1):E89-95.
24. Balady GJ, Williams MA, Ades PA, et al. Core components of cardiac rehabilitation/secondary prevention programs: 2007 update: a scientific statement from the American Heart Association Exercise, Cardiac Rehabilitation, and Prevention Committee, the Council on Clinical Cardiology; the Councils on Cardiovascular Nursing, Epidemiology and Prevention, and Nutrition, Physical Activity, and Metabolism; and the American Association of Cardiovascular and Pulmonary Rehabilitation. *Circulation.* May 22 2007;115(20):2675-2682.
25. American Association of Cardiovascular and Pulmonary Rehabilitation. Fast Facts for Cardiac Rehabilitation. http://www.aacvpr.org/Resources/CardiacPulmonaryRehabFundamentals/tabid/256/Default.aspx. Accessed October 17, 2011.
26. Ma J, Urizar GG, Jr., Alehegn T, Stafford RS. Diet and physical activity counseling during ambulatory care visits in the United States. *Prev Med.* Oct 2004;39(4):815-822.
27. Sigal RJ, Kenny GP, Wasserman DH, Castaneda-Sceppa C, White RD. Physical activity/exercise and type 2 diabetes: a consensus statement from the American Diabetes Association. *Diabetes Care.* Jun 2006;29(6):1433-1438.
28. Unick JL, Beavers D, Jakicic JM, et al. Effectiveness of Lifestyle Interventions for Individuals With Severe Obesity and Type 2 Diabetes: Results from the Look AHEAD trial. *Diabetes Care.* Aug 11 2011.
29. U.S. Department of Health and Human Services. Standards and Certification Criteria Final Rule: Fact Sheet. 2009; http://healthit.hhs.gov/portal/server.pt?CommunityID=3002&spaceID=48&parentname=&control=SetCommunity&parentid=&in_hi_userid=11673&PageID=0&space=CommunityPage.
