# Supplementary material for: Effectiveness of a Community-Based Structured Physical Activity Program for Adults With Type 2 Diabetes: A Randomized Clinical Trial
Source: JAMA Netw Open. 2022 Dec 21;5(12):e2247858. doi: 10.1001/jamanetworkopen.2022.47858 (PMC9857601; doi:10.1001/jamanetworkopen.2022.47858)
Supplement: Supplement 2. — eTable 1. Comparison of Baseline Participant Characteristics: 50% Exercise Adherence vs Nonadherence by Study Group (Per-Protocol Population) eTable 2. Baseline, Follow-up, and Changes in Clinical Values in the ITT Population eTable 3. Baseline, Follow-up, and Changes in Clinical Values in the PP Population eTable 4. Exercise Intervention Data for Individuals Who Met the Per-Protocol Criteria (N = 192) [file jamanetwopen-e2247858-s002.pdf]

Supplemental Online Content

Mukherji AB, Lu D, Qin FF, et al. Effectiveness of a community-based structured physical activity program for adults with type 2 diabetes: a randomized clinical trial. *JAMA Netw Open*. 2022;5(12):e2247858. doi:10.1001/jamanetworkopen.2022.47858

eTable 1. Comparison of Baseline Participant Characteristics: 50% Exercise Adherence vs Nonadherence by Study Group (Per-Protocol Population)
eTable 2. Baseline, Follow-up, and Changes in Clinical Values in the ITT Population
eTable 3. Baseline, Follow-up, and Changes in Clinical Values in the PP Population
eTable 4. Exercise Intervention Data for Individuals Who Met the Per-Protocol Criteria (N = 192)

This supplemental material has been provided by the authors to give readers additional information about their work.

© 2022 Mukherji AB et al. *JAMA Network Open*.

eTable 1. Comparison of Baseline Participant Characteristics: 50% Exercise Adherence vs Nonadherence by Study Group (Per-Protocol Population)

	3x Weekly Exercise				1x Weekly Exercise			
	50% exercise adherence N=58	Non-adherence N=61	ASD	*P* Value[a]	50% exercise adherence N=65	Non-adherence N=54	ASD	*P* Value[a]
Age (years), median (q1, q3)	59.8 (51.8, 64.1)	57.8 (48.9, 65.8)	0.16	.45	58.9 (54.0, 67.8)	54.6 (48.4, 61.8)	0.42	.03
Sex, No. (%)			0.12	.50			0.44	.02
Female	24 (41.4)	29 (47.5)			19 (29.2)	27 (50.0)		
Male	34 (58.6)	32 (52.5)			46 (70.8)	27 (50.0)		
Race/ethnicity[b], No. (%)			0.36	.45			0.75	.005
African American/Black	3 (5.2)	7 (11.7)			7 (10.8)	4 (7.5)		
Asian	23 (39.7)	15 (25.0)			36 (55.4)	14 (26.4)		
Hispanic or Latino	8 (13.8)	9 (15.0)			4 (6.2)	13 (24.5)		
Non-Hispanic White	19 (32.8)	23 (38.3)			13 (20.0)	15 (28.3)		
Other race[c]	5 (8.6)	6 (10.0)			5 (7.7)	7 (13.2)		
Education level, No. (%)			0.33	.41			0.38	.26
High School/GED or less	2 (3.4)	0 (0.0)			4 (6.2)	6 (11.1)		
Some college or associate degree	11 (19.0)	17 (27.9)			18 (27.7)	20 (37)		
Bachelor's Degree	21 (36.2)	21 (34.4)			20 (30.8)	17 (31.5)		
Graduate Degree	24 (41.4)	23 (37.7)			23 (35.4)	11 (20.4)		
Alcohol use[b], No. (%)			0.48	.10			0.44	.10
Never	10 (17.5)	16 (26.7)			19 (29.2)	9 (17.3)		
Rarely	30 (52.6)	18 (30.0)			19 (29.2)	21 (40.4)		
Once or less per week	12 (21.1)	17 (28.3)			18 (27.7)	19 (36.5)		
2 or more times per week	5 (8.8)	9 (15.0)			9 (13.8)	3 (5.8)		
Tobacco use[b], No. (%)			0.18	.66			0.24	.25
Never	47 (81.0)	45 (73.8)			52 (80.0)	37 (71.2)		
Former smoker	8 (13.8)	12 (19.7)			10 (15.4)	13 (25.0)		
Current smoker	3 (5.2)	4 (6.6)			3 (4.6)	2 (3.8)		

© 2022 Mukherji AB et al. *JAMA Network Open.*

	3x Weekly Exercise				1x Weekly Exercise			
**Clinical measure, median (q1, q3)**								
HbA1c, %	7.3 (6.9, 8.0)	7.2 (6.7, 8.3)	0.036	.88	7.2 (6.9, 8.0)	7.6 (7.0, 8.4)	0.24	.24
Weight, kg	87.3 (74.1, 105.9)	86.2 (78.3, 108.9)	0.21	.42	91.2 (82.0, 101.5)	92.5 (83.3, 108.8)	0.049	.54
BMI, kg/m^2	30.2 (27.7, 34.3)	31.5 (28.3, 36.8)	0.32	.15	30.2 (28.7, 35.6)	32.8 (28.6, 38.6)	0.18	.25
Waist circumference, cm	108 (100.5, 117.0)	112 (100.6, 123.0)	0.27	.21	109.0 (104.0, 119.0)	111.5 (102.0, 128.0)	0.088	.66
Systolic blood pressure (sitting), mmHg	134 (123, 141)	127 (117, 137)	0.33	.07	134 (122, 143)	129 (119, 144)	0.11	.54
Diastolic blood pressure (sitting), mmHg	84 (78, 89)	84 (78, 90)	0.03	.83	86 (79, 93)	86 (80, 94)	0.15	.41
Pulse (sitting), count per minute	75 (70, 86)	76 (70, 84)	0.01	.82	74 (67, 81)	80 (70, 86)	0.47	.02

Abbreviations: BMI, body mass index, calculated as weight in kilograms divided by height in meters squared; HbA1c, hemoglobin A1c (%)
Median (q1, q3) presented for continuous variables, and N (%) for categorical variables
[a]P-value calculated using a Chi-squared test or Fisher's exact test (for small cell counts) for categorical variables and t-test (for normal distribution values) or Mann-Whitney U test (for non-normal distribution values) for continuous measures
[b]Race/ethnicity missing or unknown N=2; alcohol use missing or decline to answer N=4; tobacco use missing or decline to answer N=2
[c]American Indian/Alaska Native, Pacific Islander, or multi-racial

© 2022 Mukherji AB et al. *JAMA Network Open.*

eTable 2. Baseline, Follow-up, and Changes in Clinical Values in the ITT Population

	3x Week Exercise	1x Week Exercise	Usual Care
**Variable**	**Mean (SE)**	**Mean (SE)**	**Mean (SE)**
HbA1c baseline	7.6 (0.1)	7.6 (0.1)	7.8 (0.1)
HbA1c 3mon	7.3 (0.1)	7.4 (0.1)	7.6 (0.2)
HbA1c 6mon	7.4 (0.1)	7.3 (0.1)	7.6 (0.1)
HbA1c baseline to 6mon f/u	-0.2 (0.1)	-0.2 (0.1)	-0.2 (0.1)
HbA1c/medication composite	37.1%	32.3%	23.8%
BMI baseline	32.5 (0.6)	33.4 (0.6)	34.4 (0.7)
BMI 3mon	32.1 (0.7)	32.5 (0.7)	34.8 (0.9)
BMI 6mon	31.9 (0.8)	32.2 (0.7)	33.3 (0.9)
BMI baseline to 6mon f/u	-0.2 (0.2)	-0.3 (0.1)	-0.1 (0.2)
Waist circumference baseline	110.6 (1.3)	113.3 (1.5)	115.6 (1.6)
Waist circumference 3mon	109.2 (1.5)	111.3 (1.6)	115.7 (1.9)
Waist circumference 6mon	109.4 (2.1)	108.7 (1.8)	112.3 (2.0)
Waist circumference baseline to 6mon f/u	-1.8 (0.7)	-1.9 (0.6)	-2.3 (0.8)
Pulse baseline	77 (1.1)	77 (1.1)	77 (1.1)
Pulse 3mon	76 (1.3)	74 (1.4)	79 (1.6)
Pulse 6mon	77 (1.4)	72 (1.5)	76 (1.5)
Pulse baseline to 6mon f/u	0 (1.1)	-1 (1.4)	-2 (1.4)
Systolic BP baseline	131 (1.4)	132 (1.6)	132 (1.6)
Systolic BP 3mon	127 (2.0)	132 (2.0)	129 (1.9)
Systolic BP 6mon	130 (2.2)	136 (2.5)	129 (2.3)
Systolic BP baseline to 6mon f/u	-1 (2.1)	3 (2.0)	-3 (2.3)
Diastolic BP baseline	85 (0.9)	86 (1.0)	86 (1.0)
Diastolic BP 3mon	82 (1.0)	84 (1.2)	83 (1.4)
Diastolic BP 6mon	82 (1.5)	84 (1.4)	84 (1.5)
Diastolic BP baseline to 6mon f/u	-2 (1.4)	0 (1.4)	-1 (1.5)

© 2022 Mukherji AB et al. *JAMA Network Open.*

eTable 3. Baseline, Follow-up, and Changes in Clinical Values in the PP Population

	3x Week Exercise	1x Week Exercise	Usual Care
**Variable**	**Mean (SE)**	**Mean (SE)**	**Mean (SE)**
HbA1c baseline	7.6 (0.1)	7.5 (0.1)	7.7 (0.1)
HbA1c 3mon	7.3 (0.1)	7.3 (0.1)	7.6 (0.2)
HbA1c 6mon	7.1 (0.1)	7.3 (0.1)	7.6 (0.1)
HbA1c baseline to 6mon f/u	-0.4 (0.1)	-0.1 (0.1)	-0.2 (0.1)
HbA1c/medication composite	41.9%	33.3%	24.2%
BMI baseline	31.6 (0.7)	32.5 (0.8)	34.0 (0.8)
BMI 3mon	31.6 (0.8)	32.4 (0.8)	34.8 (0.9)
BMI 6mon	30.7 (0.8)	32.1 (0.8)	33.3 (0.9)
BMI baseline to 6mon f/u	-0.4 (0.2)	-0.2 (0.2)	-0.1 (0.2)
Waist circumference baseline	109.0 (1.7)	111.7 (1.8)	115.6 (1.8)
Waist circumference 3mon	108.7 (2.0)	111.1 (2.0)	115.7 (1.9)
Waist circumference 6mon	106.8 (2.3)	108.4 (2.0)	112.3 (2.0)
Waist circumference baseline to 6mon f/u	-2.4 (0.9)	-1.6 (0.8)	-2.3 (0.8)
Pulse baseline	77 (1.5)	74 (1.4)	78 (1.5)
Pulse 3mon	77 (1.6)	73 (1.6)	79 (1.6)
Pulse 6mon	76 (1.7)	70 (1.6)	76 (1.5)
Pulse baseline to 6mon f/u	0 (1.1)	-2 (1.5)	-2 (1.4)
Systolic BP baseline	133 (2.0)	133 (2.2)	134 (2.1)
Systolic BP 3mon	127 (2.4)	132 (2.1)	129 (1.9)
Systolic BP 6mon	131 (2.2)	138 (2.3)	129 (2.3)
Systolic BP baseline to 6mon f/u	0 (2.3)	4 (2.3)	-3 (2.3)
Diastolic BP baseline	84 (1.4)	84 (1.5)	87 (1.3)
Diastolic BP 3mon	81 (1.2)	85 (1.4)	83 (1.4)
Diastolic BP 6mon	82 (1.4)	85 (1.4)	84 (1.5)
Diastolic BP baseline to 6mon f/u	-1 (1.3)	1 (1.4)	-1 (1.5)

© 2022 Mukherji AB et al. *JAMA Network Open.*

eTable 4. Exercise Intervention Data for Individuals Who Met the Per-Protocol Criteria (N = 192)

	Mean (SE) by Intervention Month					
	**1**	**2**	**3**	**4**	**5**	**6**
**3xWkEx (N=56)**						
Sessions/wk No.[a]	2.7 (0.1)	2.7 (0.2)	2.4 (0.2)	2.5 (0.2)	2.5 (0.2)	2.3 (0.2)
Weight Lifted (kg/wk)[b]	252.7 (85.9)	267.2 (123.6)	301.9 (147.4)	210.3 (84.5)	352.8 (175.5)	292.6 (110.4)
Aerobic Time (min/wk)[c]	73.5 (11.7)	75 (14.4)	70.8 (12.8)	83.9 (18.4)	84.4 (17.9)	90.4 (20.6)
Body Weight (kg)[d]	89.8 (2.5)	—	90.3 (2.6)	—	—	87.3 (2.8)
**1xWkEx (N=62)**						
Sessions/wk No.[a]	1.7 (0.2)	1.3 (0.1)	1.2 (0.1)	1.1 (0.1)	1.2 (0.2)	1.1 (0.2)
Weight Lifted (kg/wk)[b]	118.3 (30.9)	136.7 (42.7)	171.5 (50.3)	166.9 (70.4)	164.9 (62.1)	153.1 (56.4)
Aerobic Time (min/wk)[c]	39.2 (6.4)	40.3 (7.6)	34.8 (6.1)	32.2 (8.3)	39.2 (13.5)	40.6 (12.1)
Body Weight (kg)[d]	94 (2.6)	—	93.3 (2.8)	—	—	91.6 (2.4)
**UC (N=74)**						
Body Weight (kg)[d]	96.9 (2.4)	—	98.9 (2.6)	—	—	95.4 (2.8)

[a]Number of exercise sessions per week is the monthly mean of the average number of gym/exercise sessions attended each week for each participant (includes both gym and home sessions).
[b]Weekly average weight lifted is the monthly mean of the average weight lifted each week in strength training exercise (e.g., bicep curls, tricep curls, chest press, shoulder press, standing row, stomach crunch, squat, heel raises, etc.) for each participant.
[c]Weekly average aerobic exercise time is the monthly mean of the average time spent doing aerobic exercises (e.g., upright bike, recumbent bike, walking/jogging, dancing, treadmill, etc.) each week for each participant.
[d]Body weight in kg is from baseline, 3-months, and 6-months clinic visits since weight was not collected at the gym.

© 2022 Mukherji AB et al. *JAMA Network Open.*
