# Supplementary material for: Effectiveness of a Community-Based Structured Physical Activity Program for Adults With Type 2 Diabetes: A Randomized Clinical Trial
Source: JAMA Netw Open. 2022 Dec 21;5(12):e2247858. doi: 10.1001/jamanetworkopen.2022.47858 (PMC9857601; doi:10.1001/jamanetworkopen.2022.47858)
Supplement: Supplement 3. — Data Sharing Statement [file jamanetwopen-e2247858-s003.pdf]

Data Sharing Statement

Mukherji. Effectiveness of a Community-Based Structured Physical Activity Program for Adults With Type 2 Diabetes. *JAMA Netw Open*. Published December 21, 2022. doi:10.1001/jamanetworkopen.2022.47858

Data
Data available: Yes
Data types: Deidentified participant data, Data dictionary
How to access data: https://purl.stanford.edu/qt149dc7800
When available: beginning date: 07-11-2022

Supporting Documents
Document types: Statistical/analytic code, Informed consent form
How to access documents: Publicly accessible via URL: https://purl.stanford.edu/qt149dc7800
When available: beginning date: 07-11-2022

Additional Information
Who can access the data: Public link
Types of analyses: For any purpose.
Mechanisms of data availability: Publicly accessible via URL: https://purl.stanford.edu/qt149dc7800
